# Genome-wide association study identifies novel risk variants from *RPS6KA1*, *CADPS*, *VARS*, and *DHX58* for fasting plasma glucose in Arab population

**DOI:** 10.1038/s41598-019-57072-9

**Published:** 2020-01-13

**Authors:** Prashantha Hebbar, Mohamed Abu-Farha, Fadi Alkayal, Rasheeba Nizam, Naser Elkum, Motasem Melhem, Sumi Elsa John, Arshad Channanath, Jehad Abubaker, Abdullah Bennakhi, Ebaa Al-Ozairi, Jaakko Tuomilehto, Janne Pitkaniemi, Osama Alsmadi, Fahd Al-Mulla, Thangavel Alphonse Thanaraj

**Affiliations:** 1 Dasman Diabetes Institute, P.O. Box 1180, Dasman, 15462 Kuwait; 2 Doctoral program in Population Health, Faculty of medicine, University of Helsinki, Helsinki, Finland; 3 Sidra Medical and Research Center, Doha, Qatar; 4 Department of Public Health, University of Helsinki, Helsinki, Finland; 5 King Hussein Cancer Center, Amman, Jordan

**Keywords:** Genome-wide association studies, Genetic markers

## Abstract

Consanguineous populations of the Arabian Peninsula, which has seen an uncontrolled rise in type 2 diabetes incidence, are underrepresented in global studies on diabetes genetics. We performed a genome-wide association study on the quantitative trait of fasting plasma glucose (FPG) in unrelated Arab individuals from Kuwait (discovery-cohort:n = 1,353; replication-cohort:n = 1,196). Genome-wide genotyping in discovery phase was performed for 632,375 markers from Illumina HumanOmniExpress Beadchip; and top-associating markers were replicated using candidate genotyping. Genetic models based on additive and recessive transmission modes were used in statistical tests for associations in discovery phase, replication phase, and meta-analysis that combines data from both the phases. A genome-wide significant association with high FPG was found at rs1002487 (*RPS6KA1*) (p-discovery = 1.64E-08, p-replication = 3.71E-04, p-combined = 5.72E-11; β-discovery = 8.315; β-replication = 3.442; β-combined = 6.551). Further, three suggestive associations (p-values < 8.2E-06) with high FPG were observed at rs487321 (*CADPS*), rs707927 (*VARS* and 2Kb upstream of *VWA7*), and rs12600570 (*DHX58*); the first two markers reached genome-wide significance in the combined analysis (p-combined = 1.83E-12 and 3.07E-09, respectively). Significant interactions of diabetes traits (serum triglycerides, FPG, and glycated hemoglobin) with homeostatic model assessment of insulin resistance were identified for genotypes heterozygous or homozygous for the risk allele. Literature reports support the involvement of these gene loci in type 2 diabetes etiology.

## Introduction

A large number of genome-wide association studies have been conducted in various populations (mostly on Europeans, Americans, and East Asians), resulting in the identification of more than 100 loci conferring susceptibility to type 2 diabetes mellitus^1–4^. Meta-analysis and genotype imputations from diverse ethnic populations help identify novel markers and causal loci. However, despite the observed high prevalence of type 2 diabetes in Arab countries^5,6^, their populations were not included in global studies.

The Arabian Peninsula is at the nexus of Africa, Europe, and Asia; and has been assumed to be an early human migration route out of Africa. Consanguineous marriage (especially among first or second cousins) is an established practice among the Arabian Peninsula population. Consanguinity results in increased homozygosity, and accumulation of deleterious recessive alleles in the gene pool, creating the potential for certain variants to become more common in these endogamous population groups; these features can influence the etiology of complex disorders^7^. Therefore, elucidating novel risk variants is realistically possible in this population.

The Kuwaiti population consists of settlers from Saudi Arabia, Iran, and other neighboring countries within the Peninsula. Such settlement and subsequent admixture shaped the genetics of the Kuwaiti population. Our earlier work showed that the Kuwaiti population is heterogeneous, but structured, and carries a large burden of homozygosity^8^. Kuwaiti population groups practice consanguineous marriage; a survey in Kuwait reported that the rate of consanguineous marriages was as high as 54% and the average inbreeding coefficient was 0.0219^9^. These practices indicate that groups live in isolation by community leading to genetic isolates in extended families and Bedouin tribes^10^. Using these small population isolates can reduce the complexity of polygenic disorders by reducing the number of loci involved in disorder etiology^11^. In the present study, we performed a genome-wide association study (GWAS) on native Arab individuals from Kuwait to delineate novel risk variants for fasting plasma glucose (FPG). We further examined associations between glucose-related traits and insulin resistance traits in individuals with genotypes, heterozygous or homozygous, for the risk allele at the identified risk variants.

## Results

### Marker and sample sets

Quality control analyses resulted in a marker set of 632,375 SNPs (reduced down from 730,525), discovery cohort of 1,353 samples (reduced down from 1913), and replication cohort of 1,176 samples. The discovery cohort was estimated to have 80% power to detect associations (under additive and recessive models) with a genetic effect that explained 0.6% of the variance in the trait. The acceptable effect sizes at different allele frequencies for associations with FPG (in discovery phase) are presented in Supplementary Table S1.

### Characteristics of study participants

The study cohorts were described in our previous reports^12,13^. Participants (comprising almost equal proportions of men and women) were largely middle-aged (mean age in discovery cohort, 46.8 ± 13.8 years) (Table 1) and were largely obese (mean body mass index, 32.4 ± 7.4 kg/m^2^) with high waist circumference (102.21 ± 16.35 cm). The proportions of participants afflicted with type 2 diabetes from the discovery and replication cohorts were 45% and 39%, respectively. A total of 216 of the participants from the discovery cohort were being administered glucose-lowering medication. Mean FPG values in the discovery and replication cohorts were 7.3 ± 3.57 and 5.86 ± 2.27 mmol/L, respectively, and were in the range of the ADA-defined threshold of 5.5–6.9 mmol/L for diagnosing impaired fasting glucose. Mean HbA1c values in the discovery and replication cohorts were 7.1 ± 2.1%, and 6.00 ± 1.4%, respectively. While FPG measurements were available for all participants of the discovery cohort, HbA1c values were available for only 750; hence, markers associated with only HbA1c were excluded from further analyses.

**Table 1:** Demographic characteristics of the study participants.

	Discovery Cohort (mean ± SD)	Replication Cohort (mean ± SD)	p-values for differences between Discovery and Replication cohorts
Sex, *Male:Female*	667:686	673:503	7.96E-05
Age, *years* ± *SD*	47 ± 13.8	47 ± 10.7	0.97
Weight, *Kg* ± *SD*	88.5 ± 21.1	92.4 ± 17	3.62E-06
Height, *cm* ± *SD*	165 ± 9.6	166.5 ± 8.9	0.006
BMI, *Kg/m*^*2*^ ± *SD*	32.4 ± 7.4	31.2 ± 5.7	6.15E-06
WC, *cm* ± *SD*	102.2 ± 16.4	100.5 ± 12.1	0.003
LDL, *mmol/L* ± *SD*	3.1 ± 0.97	3.4 ± 0.9	<2.2E-16
HDL, *mmol/L* ± *SD*	1.1 ± 0.4	1.1 ± 0.3	0.82
TC, *mmol/L* ± *SD*	4.9 ± 1.1	5.2 ± 1.0	7.77E-12
TG, *mmol/L* ± *SD*	1.7 ± 1.2	1.6 ± 1.0	0.002
HbA1c, *mmol/L* ± *SD*	7.1 ± 2.1	6.0 ± 1.4	<2.2E-16
FPG, *mmol/L* ± *SD*	7.3 ± 3.6	5.9 ± 2.3	<2.2E-16
SBP, *mmHg* ± *SD*	128 ± 17.5	129.1 ± 16.7	0.06
DBP, *mmHg* ± *SD*	77.9 ± 10.6	78.7 ± 11.1	0.035
Proportion of the participants that are obese^@^ (BMI ≥ 30 *Kg/m*^2^)	59.3%	45.5%	7.43E-05
Proportion of the participants that are diabetic	44.7%	38.4%	0.002
Proportion of the participants that are hypertensive	44.9%	35.7%	3.61E-06
Proportion of the participants that consume lipid lowering medication	9.8%	0.3%	<2.2E-16
Proportion of the participants that consume glucose lowering medication	16.0%	4.6%	<2.2E-16
Proportion of the participants that consume blood pressure medication	11.9%	7.2%	0.0

Abbreviations: WC, waist circumference; TC, total cholesterol; HbA1c, glycated hemoglobin; FPG, fasting plasma glucose; SBP, systolic blood pressure; DBP, diastolic blood pressure; SD, standard deviation.

^**@**^The distribution of the participants onto normal weight (BMI 20 to <25): overweight (BMI 25 to <30): obese (BMI 30 to <40): morbid obese (BMI ≥ = 40) = 222:328:597:206 in the discovery cohort; and 93:442:559:82 in the replication cohort.

Scatterplots presenting the first three principal components derived from a merged data set of the discovery cohort and representative populations from the Human Genome Diversity Project (HGDP) are presented in Supplementary Figure S1; the scatterplots depict three genetic substructures and agree with the PCA plot (reproduced in Supplementary Figure S2**)** that we derived earlier using a set of native Kuwaiti individuals whose Arab ethnicity was confirmed through surname lineage analysis^8^.

### Associations observed in discovery and replication phases

**Figure 1:**
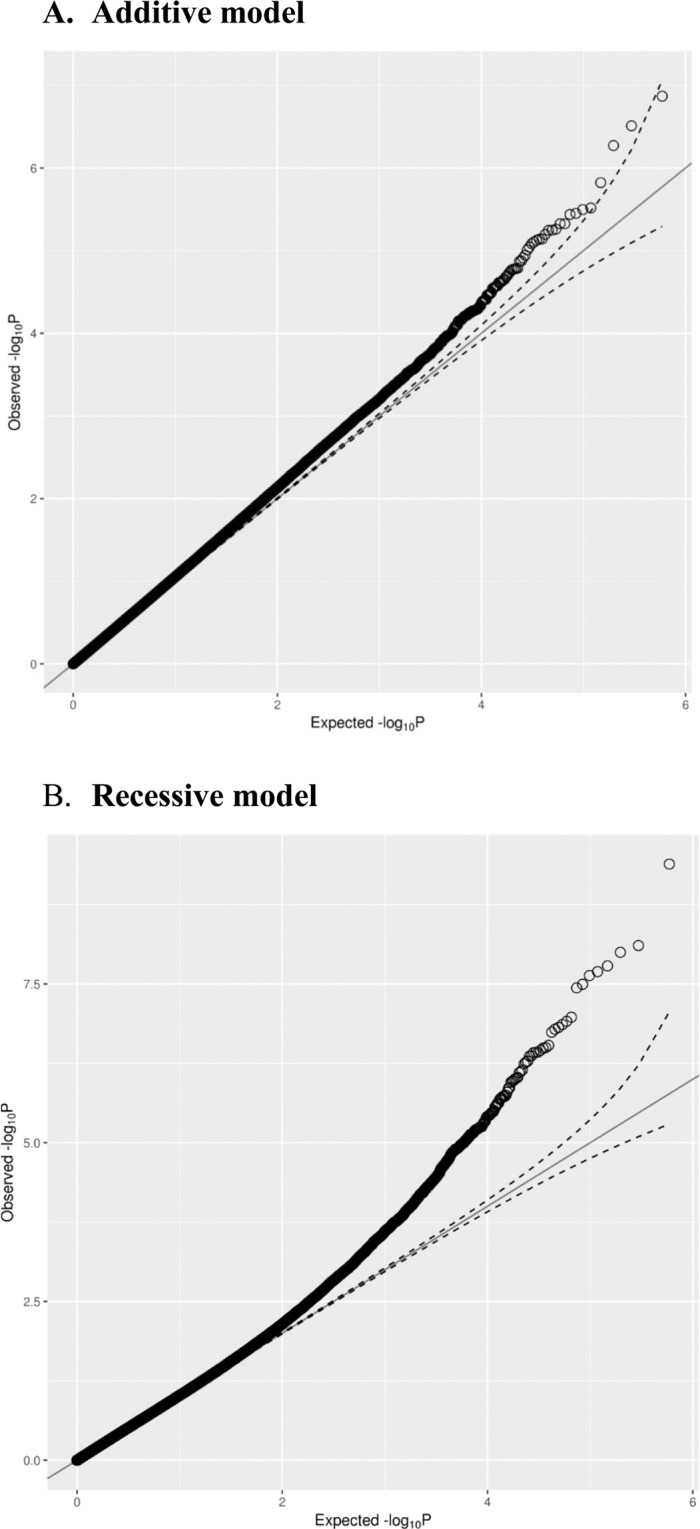
Quantile–quantile plots of the expected and observed −log_10_(***p***-values) for the association of markers with FPG under additive (λ = 1.077) and recessive (λ = 1.047) models upon regular correction.

Upon examining the association test results from discovery phase for at least nominal p-values of <1.0E-05 and acceptable beta values, we short-listed 22 markers (21 associated with FPG and 1 with both FPG and HbA1c) to carry forward to the replication phase; Table 2 presents their quality assessment values in the replication phase. Intensity maps displaying the quality of the three called genotypes at these markers are presented in Supplementary Figure S3. Quantile–quantile plots depicting the expected and observed −log_10_(*p*-values) for association of the markers with FPG are presented in Fig. 1. Genomic-control inflation factors for FPG were (λ = 1.047, recessive model; λ = 1.077, additive model**)** in tests with regular corrections and (λ = 1.031, recessive model; λ = 1.069, additive model) in tests corrected further for glucose-lowering medication. Similar values were obtained for HbA1c. These values at close to 1.0 and differing only over a small range of 1.03–1.08 do not necessitate correcting association statistics for genomic-control inflation. Manhattan plots depicting the −log_10_(*p*-values) from the GWAS for the FPG trait are presented in Supplementary Figure S4. Four markers (i.e., rs12488539, rs6762914, rs1199028, rs7329697) failed the SNP quality assessment tests for Hardy–Weinberg equilibrium quality control (HWE >10^−6^); and none failed the test for allele frequency consistency (between discovery and replication phases). Table S2 lists, for all 22 markers, results of association tests (with regular corrections and additionally corrected for diabetes medication) from the discovery and replication phases as well as meta-analysis of the combined results from both phases. The analysis produced a short-list of four associations for FPG that showed significant p-values in discovery phase (one at a genome-wide significant p-value of <1.8E-08 and three at nominal p-values of <1.0E-05) and that passed the p-value threshold in the replication phase; three of them reached genome-wide significance in the meta-analysis that combines and jointly analyze the data from both the discovery and replication phases (Table 3). Such markers were rs1002487/[intronic from *RPS6KA1*] (p-discovery = **1.64E**-**08**, p-replication = 3.71E-04, p-combined = **5.72E**-**11**), rs487321/[intronic from *CADPS*] (p-discovery = 1.53E-07, p-replication = 2.25E-06, p-combined = **1.83E**-**12**), rs707927/[intronic from *VARS* and 2 Kb upstream of *VWA7*] (p-discovery = 8.24E-06, p-replication = 8.25E-05, p-combined = **3.07E**-**09**), and rs12600570/[intronic from *DHX58*] (p-discovery = 7.49E-06, p-replication = 4.67E-03, p-combined = 2.72E-07); the former two were recessive and the latter were additive markers. Further corrections for glucose-lowering medication retained significant p-values and effect sizes. Upon performing inverse normal transformation on the FPG traits, p-values for the association of rs707927 improved to 1.26E-07 (effect size = 0.33). The *RPS6KA1* marker was also associated with HbA1c at close to the p-value threshold for genome-wide significance (p-discovery = 4.91E-08; p-replication = 2.71E-03; p-combined = 7.27E-09).

**Table 2:** SNP quality assessment statistics for the 22 markers assessed in the replication phase.

Chr	SNP	Ref/Alt Allele, Trait^model^	Discovery					Replication				
			EAF	Genotype	O(HET)	E(HET)	p-value	EAF	Genotype	O(HET)	E(HET)	p-value
1	rs1002487	T/C, FPG, HbA1C^#^	0.0594	5/151/1196	0.1117	0.112	0.8088	0.05119	5/110/1057	0.09386	0.0972	0.2241
2	rs4143782	C/T, FPG^@^	0.1812	47/396/909	0.1117	0.112	0.8088	0.1702	35/330/811	0.2803	0.2824	0.7791
3	rs12488539^*&*^	G/T, FPG^@^	0.2914	110/565/672	0.2929	0.2967	0.6466	*0.2047*	0/481/695	0.4094	0.3256	*1.26E-16*^*&*^
3	rs6762914^*&*^	T/C, FPG^@^	0.3197	135/595/623	0.4195	0.413	0.5978	*0.205*	0/482/694	0.4101	0.326	*5.5E-11*^*&*^
3	rs487321	A/G, FPG^#^	0.0821	8/206/1138	0.1524	0.1507	0.8567	0.0564	7/118/1048	0.1004	0.1066	0.0414
5	rs17065898	T/C, FPG^@^	0.1949	55/413/874	0.4398	0.435	0.708	*0.0959*	14/201/961	0.1709	0.1756	0.292
6	rs707927	A/G, FPG^@^	0.1062	15/257/1079	0.3077	0.3138	0.4864	0.1014	22/193/958	0.1649	0.1823	0.0121
6	rs1145784	G/A, FPG^#^	0.0983	12/242/1099	0.1902	0.1899	1	0.09617	16/194/965	0.1651	0.1738	0.09178
7	rs2522219	A/G, FPG^#^	0.04922	4/125/1222	0.1789	0.1773	0.8781	0.03712	0/87/1085	0.07423	0.0715	0.4022
8	rs1199028^*&*^	A/C, FPG^#^	0.1478	28/342/976	0.09252	0.0936	0.5619	0.1943	58/238/615	0.2613	0.3131	*2.3E-06*^*&*^
8	rs2599723	G/A, FPG^#^	0.0518	4/132/1214	0.09778	0.09833	0.7794	0.0627	7/134/1032	0.1144	0.1176	0.262
10	rs3812689	G/A, FPG^#^	0.06135	10/146/1197	0.2541	0.252	0.8291	0.0664	6/144/1024	0.1227	0.1241	0.6371
11	rs918988	T/C, FPG^@^	0.4217	256/629/468	0.4649	0.4877	0.0842	*0.3236*	165/598/671	0.417	0.4377	0.0855
11	rs1151501	A/G, FPG^@^	0.1116	16/270/1067	0.1996	0.1983	0.8917	0.0889	15/179/979	0.1527	0.162	0.0339
12	rs11179003	C/T, FPG^#^	0.0565	9/135/1209	0.0997	0.1067	0.0342	0.03731	3/101/1330	0.0704	0.0718	0.4451
13	rs7329697^*&*^	T/C, FPG^#^	0.09904	13/242/1098	0.1789	0.1785	1	0.113	41/184/951	0.1565	0.2005	*5.2E-09*^*&*^
13	rs4646213	G/A, FPG^#^	0.09202	12/225/1116	0.1663	0.1671	0.8702	0.09327	11/197/966	0.1678	0.1691	0.7305
14	rs3784240	G/A, FPG^#^	0.06615	11/157/1185	0.116	0.1235	0.04233	0.05641	6/120/1044	0.1026	0.1065	0.2609
15	rs1256826	A/G, FPG^@^	0.1135	20/267/1066	0.1973	0.2012	0.498	0.1213	22/240/909	0.205	0.2131	0.2151
17	rs930514	A/G, FPG^@^	0.4933	331/671/349	0.4967	0.4999	0.8277	0.4801	271/581/321	0.4944	0.4992	0.7114
17	rs12600570	C/T, FPG^@^	0.1482	34/333/986	0.2461	0.2525	0.3341	0.1444	28/358/1048	0.2497	0.247	0.7491
18	rs9959376	C/T, FPG^#^	0.09726	20/223/1109	0.1649	0.1756	0.02979	0.0966	18/191/967	0.1625	0.1745	0.0142

^#^Association with the trait was observed under the genetic model based on recessive mode of inheritance; ^**@**^association with the trait was observed under the genetic model based on additive mode of inheritance.

^&^The markers (rs12488539, rs6762914, rs1199028 and rs7329697) fail in HWE test in replication phase.

**Table 3:** List of the four identified risk variants associated with FPG either at genome-wide significant p-values (<1.8E-08) or at nominal p-values of 1.0 < E-06.

SNP: Effect Allele: Trait	Gene: functional consequences	Phase	Effect Size^R^	P-value^R^	Effect Size^DM^	P-value^DM^
rs1002487: C^#^, FPG	*RPS6KA1*: intronic	Discovery	8.315	1.64E-08	8.297	1.58E-08
		Replication	3.442	3.7E-04	3.509	2.15E-04
		Meta	6.551	5.72E-11	6.652	2.89E-11
rs487321: A^#^, FPG	*CADPS*: intronic	Discovery	6.133	1.53E-07	6.161	1.23E-07
		Replication	3.955	2.25E-06	3.88	3.033E-06
		Meta	7.047	1.83E-12	7.031	2.054E-12
rs707927: G^@,$^, FPG	*VARS*, *VWA7*: intron in *VARS*, 2 Kb upstream of *VWA7*	Discovery	0.9453	8.24E-06	0.9262	1.19E-05
		Replication	0.6375	8.25E-05	0.6503	3.18E-05
		Meta	5.928	3.074E-09	6.033	1.61E-09
rs12600570: T^@^, FPG	*DHX58*: intronic	Discovery	0.8166	7.49E-06	0.8374	4.11E-06
		Replication	0.3892	4.67E-03	0.3682	5.65E-03
		Meta	5.142	2.715E-07	5.186	2.15E-07
The following associations with HbA1c are shown in this table for the sake of completion; HbA1c associations are not considered significant except in the case of *RPS6KA1*.						
rs1002487: C^#^, HbA1C	*RPS6KA1*: intronic	Discovery	7.367	4.91E-08	7.186	9.649E-08
		Replication	1.811	2.71E-03	1.875	0.00115
		Meta	5.784	7.27E-09	5.896	3.71E-09
rs487321: A^#^, HbA1C	*CADPS*: intronic	Discovery	2.387	2.47E-03	2.38	2.44E-03
		Replication	1.893	2.77E-04	1.826	3.82E-04
		Meta	4.723	2.32E-06	4.569	3.18E-06
rs707927: G^@^, HbA1C	*VARS*, *VWA7*: intron in *VARS*, 2 Kb upstream of *VWA7*	Discovery	0.5632	5.43E-04	0.5502	6.96E-04
		Replication	0.3689	1.63E-04	0.3799	8.33E-05
		Meta	5.088	3.61E-07	5.181	2.21E-07
rs12600570: T^@^, HbA1C	*DHX58*: intronic	Discovery	0.31	2.82E-02	0.3344	1.76E-02
		Replication	0.194	1.98E-02	0.1805	2.81E-02
		Meta	3.179	1.47E-03	3.178	1.48E-03

^**EffectSize**^, Effect size represents beta value for discovery and replication phases, and Z-score for meta-analysis. R-regular correction: Corrected for age, sex and the top 10 principal components that resulted from the Principal Components Analysis of the genotype data; DM: Corrected for diabetes medication in addition to the regular correction.

^**#**^association with the trait was observed under the genetic model based on recessive mode of inheritance; ^**@**^association with the trait was observed under the genetic model based on additive mode of inheritance.

^**$**^Upon performing inverse normal transformation on the FPG values, the p-values for association of the marker rs707927 with FPG improved in the discovery phase; the values were (p-value = 1.26E-07; effect size = 0.3314) which upon further correction for diabetes medication were (p-value = 2.72E-07; effect size = 0.3226).

### Considering the diabetes and obesity status of the participants as covariates for adjustments on the association models

45% of participants in the discovery phase, and 38% of participants in the replication phase, respectively, were diagnosed for diabetes (see Table 1). It is further the case that obesity seems to be a major driver of diabetes in the whole sample – 59% of participants in the discovery phase and 46% of participants in the replication phase, respectively, were obese. Thus, it is important to perform corrections for the association tests for diabetes and obesity status along with corrections for diabetes and lipid lowering medications (lipid lowering medications can influence FPG levels). Upon performing the corrections for these 4 covariates along with the regular corrections, it was found that the p-values remained significant at p-combined = 1.38E-10 (for *RPS6KA1* marker), 1.88E-13 (for *CADPS* marker), 1.23E-08 (for *VARS* marker) and 2.78E-05 (for *DHX58* marker) (Table 4).

**Table 4:** Results from the analysis of correcting the observed associations for the additional covariates of obesity and diabetes status of the participants.

SNP: Effect Allele: Trait	Gene	Phase	Effect Size^BMI^	P-value^BMI^	Effect Size^LM^	P-value^LM^	Effect Size^DS^	P-value^DS^	Effect Size ^DS+BMI+DM+LM^	P-value^DS+BMI+DM+LM^
rs1002487: C^#^, FPG	*RPS6KA1*: intronic	Discovery	8.416	1.02E-08	8.379	1.23E-08	6.388	1.53E-07	6.482	1.01E-07
		Replication	3.355	2.62E-03	3.442	3.73E-04	3.492	2.16E-04	3.46	2.10E-04
		Meta	6.233	4.58E-10	6.233	4.56E-10	6.357	2.05E-10	6.418	**1.38E-10**
rs487321: A^#^, FPG	*CADPS*: intronic	Discovery	6.145	1.35E-07	6.177	1.22E-07	4.468	3.83E-06	4.48	3.61E-06
		Replication	3.904	2.59E-06	3.979	2.06E-06	3.994	1.28E-08	4.00	8.97E-09
		Meta	7.042	1.90E-12	7.089	1.35E-12	7.303	2.81E-13	7.356	**1.88E-13**
rs707927: G^@,$^, FPG	*VARS*, *VWA7*: intron in *VARS*, 2 Kb upstream of *VWA7*	Discovery	0.9289	1.14E-05	0.931	1.12E-05	0.658	1.73E-04	0.6523	2.01E-04
		Replication	0.6446	6.29E-05	0.683	2.64E-05	0.614	6.59E-06	0.584	1.51E-05
		Meta	5.926	3.11E-09	6.073	1.25E-09	5.851	4.87E-09	5.696	**1.23E-08**
rs12600570: T^@^, FPG	*DHX58*: intronic	Discovery	0.7914	1.42E-05	0.831	5.19E-06	0.530	4.44E-04	0.5092	7.83E-04
		Replication	0.3757	5.20E-03	0.391	3.88E-03	0.309	6.59E-03	0.291	9.74E-03
		Meta	5.025	5.05E-07	5.238	1.62E-07	4.392	1.12E-05	4.191	**2.78E-05**

^#^Association with the trait was observed under the genetic model based on recessive mode of inheritance; ^**@**^association with the trait was observed under the genetic model based on additive mode of inheritance.

^**EffectSize**^Effect size represents beta value for discovery and replication phases, and Z-score for meta-analysis. ^**R**^Regular correction - Corrected for age, sex and the top 10 principal components that resulted from the Principal Components Analysis of the genotype data; ^**BM**^I,Corrected for BMI in addition to the regular correction; ^**LM**^Corrected for lipid medication in addition to the regular correction; ^**DS**^Corrected for diabetes status in addition to the regular correction; ^**BMI+LM+DM**^Corrected for BMI and lipid & diabetes medications in addition to the regular correction.

### Sensitivity analysis

A concern arises as to whether the FPG values measured in individuals receiving glucose-lowering medication represent “naturally” observed values in the population. We addressed this concern by way of performing a sensitivity analysis to add a value of 2.5 mmol/L to the FPG values of the participants taking diabetes medication and then performing the association tests; the value of 2.5 mmol/L is an average effect size (p-value < 0.001) that we observed in an in-house clinical database of diabetic patients visiting clinics in our institute. The results of association tests with the preadjusted FPG values for the four identified associations (with corrections for regular confounders and BMI) are presented in Table 5. The associations retained the p-values.

**Table 5:** Results from sensitivity analysis of preadjusting the FPG measurements by a fixed value (2.5 mmol/L) per diabetes medication status.

SNP: Effect Allele: Trait	Gene	Phase	Effect Size^R^	P-value^R^	Effect Size^BMI^	P-value^BMI^
rs1002487: C^#^, FPG	*RPS6KA1*: intronic	Discovery	8.371	7.63E-08	8.48	4.78E-08
		Replication	3.378	1.27E-03	3.43	9.29E-04
		Meta	4.895	9.85E-07	6.201	5.59E-10
rs487321: A^#^, FPG	*CADPS*: intronic	Discovery	6.041	1.01E-06	6.055	8.94E-07
		Replication	4.163	6.11E-06	4.092	7.35E-06
		Meta	6.396	1.59E-10	6.645	3.04E-11
rs707927: G^@,$^, FPG	*VARS*, *VWA7*: intron in *VARS*, 2 Kb upstream of *VWA7*	Discovery	1.011	6.34E-06	0.9928	8.93E-06
		Replication	0.6265	4.49E-04	0.5916	8.38E-04
		Meta	5.674	1.39E-08	5.502	3.75E-08
rs12600570: T^@^, FPG	*DHX58*: intronic	Discovery	0.9928	8.93E-06	0.7241	1.7E-04
		Replication	0.4363	3.30E-03	0.4233	4.1E-03
		Meta	4.835	1.33E-06	4.803	1.56E-06

### Assessing the identified associations in sub-cohorts of entirely diabetic or of entirely non-diabetic participants

The discovery and replication cohorts used in this study included both diabetic patients and healthy participants; as mentioned above, the identified associations retained significance when the models were adjusted for the covariate of diabetes status. It is often the case that quantitative trait associations are done on entirely non-diabetic participants or on entirely diabetic patients (which gives a higher chance of translating the findings to clinical utility). We distributed the discovery cohort (n = 1353) and replication cohort (n = 1176) onto four sub-cohorts: (i) Discovery_diabetic (n = 605); (ii) Discovery_non-diabetic (n = 748); (iii) Replication_diabetic (n = 452); and (iv) Replication_non-diabetic (n = 724). We performed association tests with each of the four sub-cohorts followed by three meta-analysis (Meta_diabetic: combining results from Discovery_diabetic and Replication_diabetic), (Meta_non-diabetic: combining results from Discovery_non-diabetic and Replication_non-diabetic) and (Meta_all: combining results from all the four sub-cohorts). With regular corrections performed on the association tests, the effect sizes and p-values remained significant in the Meta_diabetic analysis (Table 6) for the markers from the *RPS6KA1* (β = 6.01; p = 1.84E-09)*, CADPS* (β = 5.13; p = 2.86E-07) and *VARS* (β = 4.68; p = 2.83E-06) genes and in the Meta_non-diabetic analysis for the marker from the *DHX58* gene (β = 3.81; p = 1.30E-04); considering that the sizes of the sub-cohorts reduced considerably, these values can be considered significant. In addition, the p-values for Meta_all analysis (β = 5.46; p = 4.82E-08) remained significant for the *VARS* marker.

**Table 6:** Results from the analysis of examining the identified associations in sub-cohorts of entirely diabetic patients or of entirely healthy participants.

SNP: Effect Allele: Trait	Gene: functional consequences	Phase	Effect Size^R^	P-value^R^	Effect Size^BMI+LM^	P-value^BMI+LM^	Effect Size^BMI+LM+DM^	P-value^BMI+LM+DM^
rs1002487: C^#^, FPG	*RPS6KA1*: intronic	Discovery_diabetic	6.396	2.48E-04	6.487	2.11E-04	6.488	2.12E-04
		Discovery_non- diabetic^&^	NA	NA	NA	NA		
		Replication_diabetic	17.83	4.74E-07	17.72	6.07E-07	17.7	6.50E-07
		Replication_non- diabetic	0.0086	0.9886	−0.08115	0.8861		
		**Meta_diabetic**	**6.011**	**1.84E-09**	**6.014**	**1.81E-09**	**6.004**	**1.93E-09**
		Meta_non-diabetic	0.014	0.9886	0.143	0.8861		
		Meta_all	4.173	3.0E-05	4.062	4.86E-05		
rs487321: A^#^, FPG	*CADPS*: intronic	Discovery_diabetic	5.781	3.1E-04	5.799	3.1E-04	5.797	3.1E-04
		Discovery_non- diabetic	−0.117	0.9872	0.1009	0.8885		
		Replication_diabetic	9.392	2.1E-04	9.346	2.36E-04	9.594	1.84E-04
		Replication_non- diabetic	2.479	4.64E-08	2.429	1.07E-08		
		**Meta_diabetic**	**5.132**	**2.86E-07**	**5.116**	**3.12E-07**	**5.154**	**2.55E-07**
		Meta_non-diabetic	4.190	2.78E-05	4.486	7.27E-06		
		Meta_all	6.420	1.36E-10	6.648	2.97E-11		
rs707927: G^@^, FPG	*VARS*, *VWA7*: intron in *VARS*, 2 Kb upstream of *VWA7*	Discovery_diabetic	1.153	1.50E-03	1.157	1.51E-03	1.155	1.54E-03
		Discovery_non- diabetic	0.1979	3.3E-02	0.1858	4.18E-02		
		Replication_diabetic	1.516	4.23E-04	1.518	4.35E-04	1.517	4.47E-04
		Replication_non- diabetic	0.2364	1.1E-02	0.2011	2.04E-02		
		**Meta_diabetic**	**4.683**	**2.83E-06**	**4.677**	**2.91E-06**	**4.668**	**3.05E-06**
		Meta_non-diabetic	3.327	8.78E-04	3.083	2.05E-03		
		Meta_all	5.458	4.82E-08	5.259	1.45E-07		
rs12600570: T^@^, FPG	*DHX58*: intronic	Discovery_diabetic	0.8421	8.46E-03	0.8328	9.55E-03	0.8303	9.85E-03
		Discovery_non- diabetic	0.2308	3.35E-03	0.217	5.21E-03		
		Replication_diabetic	0.5816	0.101	0.5767	0.1052	0.5874	0.1002
		Replication_non- diabetic	0.1955	1.2E-02	0.1955	1.17E-02		
		**Meta_diabetic**	**3.080**	**2.07E-03**	**3.035**	**2.4E-03**	**3.042**	**2.35E-03**
		Meta_non-diabetic	3.814	1.30E-04	3.724	1.96E-04		
		Meta_all	4.898	9.71E-07	4.799	1.59E-06		

^&^In the case of the *RPS6KA1* marker, all the individuals with genotype homozygous for risk allele were seen with the sub-cohort of Discovery_diabetic) and hence results for Discovery_ non-diabetic sub-cohort were unavailable.

^#^Association with the trait was observed under the genetic model based on recessive mode of inheritance; ^**@**^association with the trait was observed under the genetic model based on additive mode of inheritance.

^**EffectSize**^Effect size represents beta value for discovery and replication phases, and Z-score for meta-analysis. ^**R**^Regular correction - Corrected for age, sex and the top 10 principal components that resulted from the Principal Components Analysis of the genotype data; ^**BMI+LM**^Corrected for BMI and lipid medication in addition to the regular correction; ^**BMI+LM+DM**^Corrected for BMI and lipid & diabetes medications in addition to the regular correction.

### Examining the NHGRI-EBI GWAS catalog for previous association reports on the identified risk variants

While none of the identified risk variants was associated with any trait in previous GWAS, the gene loci were often associated with traits related to diabetes: *RPS6KA1* with glucose homeostasis traits^14^, sporadic amyotrophic lateral sclerosis^15^, and the symptom of rosacea^16^; *DHX58* with coronary artery disease (CAD)^17^; *VARS* with blood plasma proteome^18^, autism spectrum disorder (ASD)^19^, and inflammatory bowel disease (IBD)^20^; *VWA7* with blood protein levels^18^, ASD^19^, and IBD^20^; and *CADPS* with treatment interaction of sulfonylurea (a glucose-lowering drug)^21^, heart failure-related metabolite levels^22^, and obsessive-compulsive symptoms^23^.

### LD markers and regional associations

**Figure 2:**
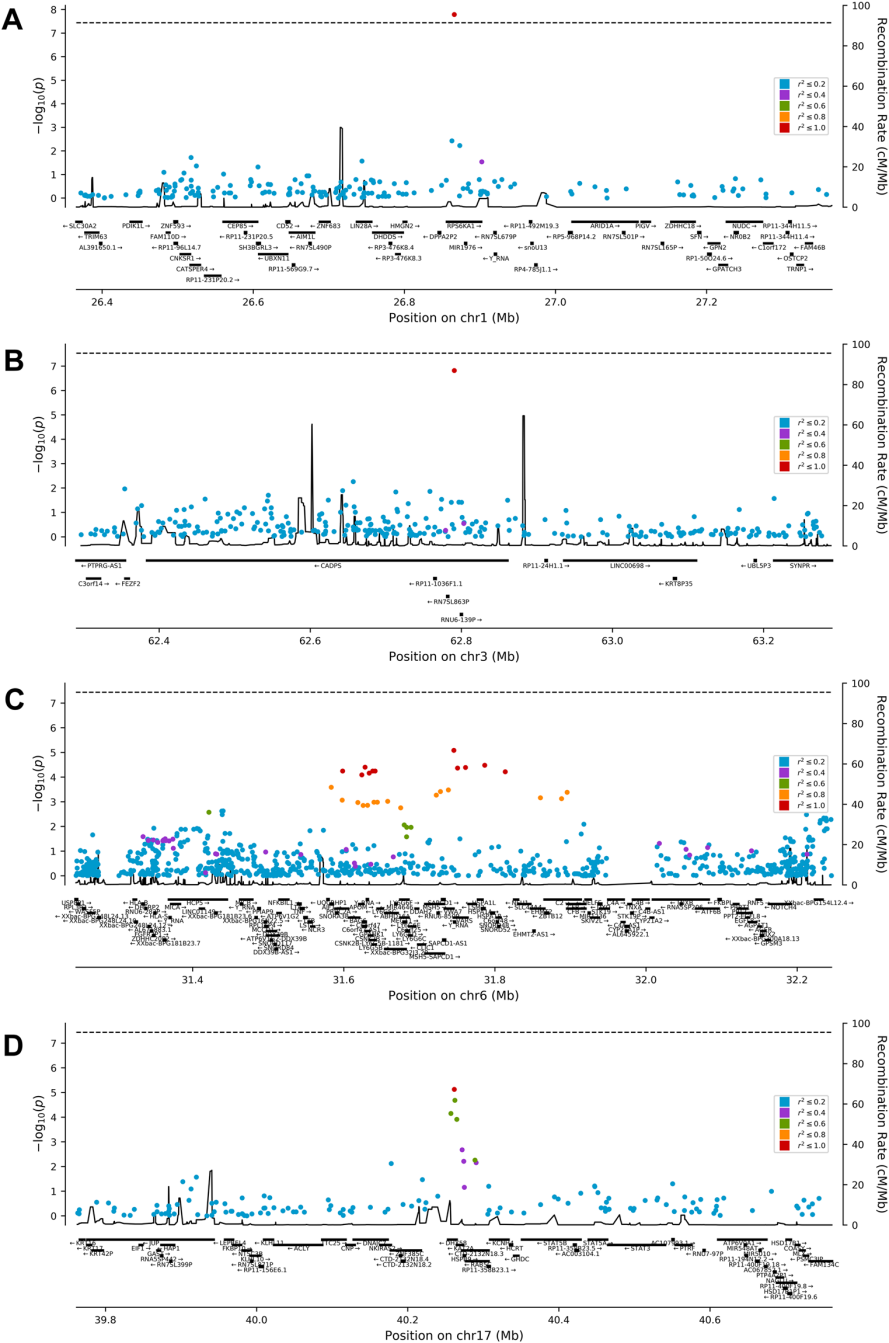
Regional association plots showing the 4 identified risk variants (**A**) rs1002487, (**B**) rs487321, (**C**) rs707927, (**D**) rs12600570) and the markers in LD (from a 500 Kb genome region centered at the risk variants) with the risk variants in their respective gene regions and their association with FPG. The SNPs are color-coded as per the r^2^ value for the SNP with the identified risk variant (Blue dots: r^2^ ≤ 0.2; Purple dots: r^2^ > 0.2 & ≤ 0.4; Green dots: r^2^ > 0.4 & ≤ 0.6; Orange dots: r^2^ > 0.6 & ≤ 0.8; Red dots: r^2^ > 0.8 & ≤ 1.0). The X-axis represents the gene region in physical order; the Y-axis represents −log_10_ P-value of the associations with FPG for all the SNPs. The dashed horizontal line represents a p-value of 3.60E-08. To generate regional association plot for a SNP-trait association, all the genotyped SNPs (passing the quality control analyses) from a region of around 500 Kb centered on the SNP were tested for association with the trait; the resultant statistics and the SNPs were displayed in the regional association plot. Region-plot tool (https://github.com/pgxcentre/region-plot) was used to produce regional plots.

Figure 2 presents regional association plots for regions of 500 Kb centered at the identified four risk variants; these regions (other than for the *CADPS* marker) were gene-dense. The (*VARS, VWA7*) and *DHX58* markers had 21 and 7 LD partners (r^2^ > 0.59), respectively. Several LD partners were associated with FPG at suggestive p-values of <1E-04 (Supplementary Table S3). Examination of the NHGRI-EBI GWAS catalog listed the following two LD partners (that associated in our study population at a p-value of E-05): (i) **rs2074158-T** (missense) (LD [r^2^ = 0.56] partner of *DHX58* risk variant) associated with CAD (p-value = 2.0E-10) in UK BioBank populations^17^; and (ii) **rs9469054-A** (intronic) (LD [r^2^ = 0.85] partner of [*VARS, VWA7*] risk variant) associated with monocyte count (p-value = 1.0E-20)^24^; shared genetic pathways linking blood cell counts with complex pathologies (including CAD) have been reported^24^.

### ROH segments overlaying the identified risk variants

All of the four reported risk variants were in ROH (Table 7). The observed maximum values for of the ROH region lengths (mean ± SD of the ROH groups) were 8 Mb (*RPS6KA1* marker), 15.5 Mb (*CADPS*), 9.9 Mb (*VARS, VWA7*), and 6.96 Mb (*DHX58*). The two recessive risk variants from *RPS6KA1* and *CADPS* were in “known” ROH segments, while the two additive markers from (*VARS*, *VWA7*) and *DHX58* were in “novel” segments. However, LD partners of the additive risk variants lay in “known” ROH segments – one such marker (i.e., rs2074158/*DHX58*) in LD with the *DHX58* risk variant is listed in the GWAS catalog as being associated with CAD (see Table 7). Presence of the identified ROH segments (to which the associated variant overlaps) is found more often in sub-cohort of diabetic participants than in sub-cohort of non-diabetic participants, though the size of the former sub-cohort (n = 605) is smaller than that of the latter sub-cohort (n = 748); however, the differences are not seen statistically significant.

**Table 7:** ROH regions overlaying the identified risk variants.

SNP	ROH group and the method used to identify the ROH^@^	Consensus ROH region	Distance to SNP from consensus ROH (in Mb)	Number of individuals from the discovery cohort (n = 1353) harboring the ROH (Distribution into sub-cohort of participants diagnosed for T2DM (n = 605) versus sub-cohort of non-diabetic participants (n = 748))	Length of consensus ROH (in Kb)	Count of SNPs in consensus ROH region	Mean ± SD of ROH groups	Distance to SNP from mean ± SD window (in Mb)	Presence of SNP in ROH regions identified in worldwide population (from Pemberton *et al*. study^48^)
rs1002487/*RPS6KA1*	S1818^1^	1:28864435–29062427	1.99	51 (29:22)	197.99	11	24917436–33009426	Overlapping	Yes
	S1557^2^	1:28056342–28084571	1.19	44 (27:17)	28.23	5	27723540–28417372	0.85	
rs487321/*CADPS*	S7177^1^	3:62647115–63435226	0.143	29 (17:12)	788.11	226	3:55304385–70777955	Overlapping	Yes
	S7176^1^	3:62315312–62315312	0.475	29 (16:13)	0.001	1	3:55748124–68882499	Overlapping	
	S4114^2^	3:61981197–62189189	0.60	31 (17:14)	207.99	85	3:56659352–67511033	Overlapping	
	S4115^2^	3:62604010–62604010	0.186	31 (16:15)	0.001	1	3:57340271–67867749	Overlapping	
	S4116^2^	3:62883050–63333375	0.0924	31 (18:13)	450.32	101	3:57761318–68455106	Overlapping	
	S4117^2^	3:63663215–63670140	6.93	31 (18:13)	0.873	3	3:58212832–69120521	Overlapping	
rs707927/[*VARS*, *VWA7*]	S1706^1^	6:31001421–32989521	0.744	53 (29:24)	1988.10	1077	6:26827255–36745549	Overlapping	No, But LD SNP rs805267 (r^2^ = 0.69) is present
	S687^2^	6:29569045–29593788	2.176	71 (38:33)	24.74	24	6:26617526–32545306	Overlapping	
	S824^2^	6:31872383–32161430	0.126	64 (34:30)	289.05	126	6:29115193–34918619	Overlapping	
	S1000^2^	6:30112623–30125537	1.619	57 (30:27)	12.91	30	6:27129747–33108413	Overlapping	
	S1001^2^	6:31572927–31572927	0.173	57 (31:26)	0.001	1	6:28490667–34655186	Overlapping	
rs12600570/*DHX58*	S5153^1^	17:39980819–40041676	Overlapping	34 (19:15)	60.858	8	17:36532212–43490282	Overlapping	No, But LD SNP rs2074158 (r^2^ = 0.56) is present
	S1741^2^	17:40041676–40063083	0.219	43 (22:21)	21.408	5	17:39559717–40545041	Overlapping	

^@^Two approaches were used to identify ROH segments (see Methods for details). Method 1: Markers that passed quality control were pruned for LD (r^2^ > 0.9) (n = 568,670) and employed to detect ROH segments using parameters suggested by Howrigan *et al*.^52^; Method 2: Un-pruned marker set (n = 632,375) was employed to detect ROH using parameters deployed in Christofidou *et al*.^53^.

### Gene expression regulation by the identified risk variants

Examination of Genotype-Tissue Expression (GTeX) data (https://www.gtexportal.org) revealed that all four risk variants regulate the expression of their own or other genes. The ***RPS6KA1*** marker regulates the *DHDDS* gene in the heart’s left ventricle; the ***CADPS*** marker regulates itself in the artery-tibial and adipose-subcutaneous tissues; the (***VARS, VWA7***) marker regulates a number of genes [*LY6G5B* (artery-tibial, testis, muscle-skeletal, thyroid); *GPANK1* (esophagus-mucosa, skin); *AIF1* (whole blood), *C6orf*2*5* (skin), *SAPCD1*-*AS1* (skin); and *TNXA* (skin)]; the ***DHX58*** marker regulates itself (in artery-tibial, adipose-subcutaneous, adipose-visceral, pancreas, and heart) and other genes such as *KCNH4* (esophagus-muscularis), *HSPB9* (testis), and *RAB5C* (adipose-subcutaneous, pancreas, muscle-skeletal).

### Associations between glucose-related traits and insulin resistance traits at the risk variants

Allelic association test statistics (Supplementary Table S4) for the identified risk variants in the third cohort of 283 samples considered for insulin resistance analysis indicated that the *RPS6KA1*, *(VARS, VWA7)*, and *CADPS* markers passed the p-value threshold (<0.05) for associations with insulin resistance traits of HOMA-IR and HOMA-β and with the glucose-related traits of FPG and HbA1c; in addition, the association of the *RPS6KA1* marker with TG was replicated.

Results from multivariate analysis to examine relationships between glucose-related (FPG, HbA1C, TG) and insulin resistance (HOMA-IR, HOMA-β, C-peptide, HOMA-S) traits in the context of observed genotypes at risk variants (Table 8) indicated possible associations of the identified risk variants with insulin resistance:

(I) *RPS6KA1 marker:* With genotypes homozygous for the risk allele, interactions between (TG, FPG and HbA1c) and insulin resistance traits (HOMA-β, C-peptide, HOMA-S) were observed at the multiple testing significance threshold of <0.003. With the heterozygous genotype, TG was associated with HOMA-S at a p-value < 0.05.
(II) *[VARS, VWA7] marker:* With genotypes that are heterozygous or homozygous for the risk allele, interactions between FPG and insulin resistance traits (HOMA-β and HOMA-S) were observed at the multiple testing significance threshold of <0.003. With a heterozygous genotype, interactions between HbA1c and insulin resistance traits (HOMA-IR and HOMA-S) were observed at the multiple testing significance threshold of <0.003. TG also interacted with HOMA-S at a p-value = 0.007 with a heterozygous genotype.
(III) *CADPS marker:* With a heterozygous genotype, associations between TG and HOMA-IR were observed at the multiple testing significance threshold of <0.003. Interaction between FPG and HOMA-β with a heterozygous genotype could be seen at a p-value < 0.003.
(IV) *DHX58 marker:* With genotypes homozygous for the risk allele, TG and FPG were seen to be associated with (HOMA-IR and C-peptide levels) and HOMA-β, respectively, at the multiple testing significance threshold of <0.003. With heterozygous genotypes, FPG was associated with both HOMA-IR and HOMA-β at p-values < 0.05.

**Table 8:** Interactions between (TG, FPG, HbA1c) and Insulin Resistance traits (HOMA-IR, HOMA-β, C-peptide, HOMA-S) with respect to genotypes at the risk variants.

Interaction	Effect Size	Std. Error	P-value^@^
**Recessive Marker rs1002487-C/RPS6KA1**			
Model: TG~rs1002487* insulin resistance traits			
CC: HOMA-IR	11.78	4.12	0.0047
TC: HOMA-IR	12.95	7.17	0.072
CC: HOMA-β	−4.31	0.69	1.5E-09
TC: HOMA-β	0.078	0.17	0.655
CC: C-peptide	1959.1	292.95	1.25E-10
TC: C-peptide	26.11	18.32	0.152
CC: HOMA-S	−2.633	0.418	1.16E-09
TC: HOMA-S	−0.299	0.145	0.0397
Model: FPG~rs1002487* insulin resistance traits			
CC: HOMA-IR	0.209	0.308	0.497
TC: HOMA-IR	−0.394	0.537	0.463
CC: HOMA-β	−0.247	0.043	3.62E-08
TC: HOMA-β	0.0013	0.011	0.900
CC: C-peptide	122.2	5.046	8.19E-07
TC: C-peptide	0.682	1.515	0.653
CC: HOMA-S	−0.162	0.032	1.25E-06
TC: HOMA-S	−0.0005	0.011	0.995
Model: HbA1C~rs1002487* insulin resistance traits			
CC: HOMA-IR	0.341	0.201	0.090
CT: HOMA-IR	−0.624	0.349	0.075
CC: HOMA-β	−0.138	0.029	3.04E-06
CT: HOMA-β	−0.0008	0.007	0.257
CC: C-peptide	66.96	14.77	8.73E-06
CT: C-peptide	−0.886	0.924	0.338
CC: HOMA-S	−0.092	0.02	1.35E-05
CT: HOMA-S	0.0037	0.007	0.606
**Additive Marker rs707927-G/*[VARS, VWA7]***			
Model: TG~rs707927* insulin resistance trait			
AG: HOMA-IR	9.845	3.351	0.0035
GG: HOMA-IR	17.318	78.544	0.825
AG: HOMA-β	−0.307	0.165	0.064
GG: HOMA-β	−0.435	0.817	0.595
AG: C-peptide	8.396	17.182	0.625
GG: C-peptide	−139.584	258.03	0.588
AG: HOMA-S	−0.313	0.116	0.007
GG: HOMA-S	0.009	0.521	0.986
Model: FPG~rs707927* insulin resistance trait			
AG: HOMA-IR	0.239	0.233	0.306
GG: HOMA-IR	24.48	5.470	1.11E-05
AG: HOMA-β	−0.034	0.009	0.00032
GG: HOMA-β	−0.194	0.047	5.17E-05
AG: C-peptide	0.100	1.272	0.937
GG: C-peptide	−77.43	19.10	6.59E-05
AG: HOMA-S	−0.020	0.008	0.012
GG: HOMA-S	−0.140	0.037	0.00019
Model: HbA1C~rs707927* insulin resistance trait			
AG: HOMA-IR	0.482	0.159	0.0024
GG: HOMA-IR	7.012	3.736	0.0615
AG: HOMA-β	−0.021	0.006	0.0013
GG: HOMA-β	−0.048	0.032	0.127
AG: C-peptide	0.827	0.788	0.295
GG: C-peptide	−22.07	11.84	0.063
AG: HOMA-S	−0.018	0.005	0.0005
GG: HOMA-S	−0.043	0.023	0.0686
**Recessive Marker rs487321-A/*CADPS***			
Model: TG~rs487321* insulin resistance trait			
GA: HOMA-IR	−17.71	6.001	0.003
AA: HOMA-IR	NA	NA	NA
GA: HOMA-β	0.091	0.186	0.623
AA: HOMA-β	NA	NA	NA
GA: C-peptide	−0.661	18.710	0.972
AA: C-peptide	NA	NA	NA
GA: HOMA-S	0.109	0.150	0.468
AA: HOMA-S	NA	NA	NA
Model: FPG~rs487321* insulin resistance trait			
GA: HOMA-IR	−0.316	0.446	0.476
AA: HOMA-IR	NA	NA	NA
GA: HOMA-β	−0.024	0.011	0.032
AA: HOMA-β	NA	NA	NA
GA: C-peptide	−1.55	1.464	0.288
AA: C-peptide	NA	NA	NA
GA: HOMA-S	0.0006	0.011	0.952
AA: HOMA-S	NA	NA	NA
Model: HbA1C~rs487321* insulin resistance trait			
GA: HOMA-IR	0.038	0.294	0.896
AA: HOMA-IR	NA	NA	NA
GA: HOMA-β	−0.007	0.007	0.330
AA: HOMA-β	NA	NA	NA
GA: C-peptide	0.088	0.881	0.920
AA: C-peptide	NA	NA	NA
GA: HOMA-S	−0.0041	0.007	0.5610
AA: HOMA-S	NA	NA	NA
**Additive Marker rs12600570-T/*DHX58***			
Model: TG~rs12600570* insulin resistance trait			
CT: HOMA-IR	−7.86	5.063	0.122
TT: HOMA-IR	59.60	17.11	0.00057
CT: HOMA-β	0.116	0.128	0.3654
TT: HOMA-β	0.667	0.490	0.1742
CT: C-peptide	3.11	17.42	0.858
TT: C-peptide	178.71	44.06	6.50E-05
CT: HOMA-S	0.155	0.093	0.095
TT: HOMA-S	−0.971	0.393	0.014
Model: FPG~rs12600570* insulin resistance trait			
CT: HOMA-IR	1.005	0.375	0.0078
TT: HOMA-IR	2.797	1.268	0.0282
CT: HOMA-β	−0.018	0.0076	0.0158
TT: HOMA-β	−0.135	0.0291	5.09E-06
CT: C-peptide	−0.781	1.391	0.574
TT: C-peptide	−10.22	3.517	0.0039
CT: HOMA-S	−0.004	0.007	-0.660
TT: HOMA-S	−0.081	0.029	-2.712
Model: HbA1C~rs12600570* insulin resistance trait			
CT: HOMA-IR	0.213	0.254	0.402
TT: HOMA-IR	0.153	0.859	0.858
CT: HOMA-β	0.002	0.0052	0.601
TT: HOMA-β	−0.012	0.0196	0.531
CT: C-peptide	−0.363	0.848	0.668
TT: C-peptide	−1.861	2.144	0.386
CT: HOMA-S	0.0015	0.004	0.720
TT: HOMA-S	−0.0176	0.0188	0.350

^@^Multiple testing significance threshold for p-value is 0.003.

All the interaction models were corrected for age and gender.

## Discussion

This study identified a novel recessive marker (rs1002487) from *RPS6KA1* (encoding Ribosomal Protein S6 Kinase A1) associated with high FPG (and HbA1c) at genome-wide significance in native Kuwaiti people of Arab descent. S6K1 signaling has distinct roles in regulating glucose homeostasis in pro-opiomelanocortin and agouti-related protein neurons, key regulators of energy homeostasis^25^; and can potentially regulate insulin resistance through phosphorylating insulin receptor substrate 1 (IRS-1)^26^. It participates in the NOTCH pathway, an effector of mTOR, and is sensitive to both insulin and certain nutrients. Our previous GWAS, using the same cohort^12^, demonstrated that the marker was also recessively associated with high TG at genome-wide significance. FPG was directly correlated with TG and inversely correlated with HDL. Adiposity, high FPG, and TG are hallmarks of insulin resistance^27^ and high FPG within the normoglycemic range can increase the risk for type 2 diabetes^28^. The presented results indicate interactions between (TG, FPG, and HbA1c) and insulin resistance traits (HOMA-β, HOMA-S, C-peptide) at multiple testing significance with genotypes homozygous for the risk allele at the risk variant; even for the heterozygous genotype, TG was associated with HOMA-S (at p-value < 0.05). Thus, the present study, reporting for the first time that the *RPS6KA1* marker is a risk variant for TG and glucose-related traits, is of considerable interest. Furthermore, in the GWAS catalog, the *RPS6KA1* gene is associated with glucose homeostasis traits, sclerosis, and the symptom of rosacea. Reports have suggested that the rare homozygous (CC) state at the marker is involved in schizophrenia^29^. The GTeX resource annotates this marker as having the potential to regulate expression of the *DHDDS* gene, a locus associated with developmental delay and seizures (with or without movement abnormalities); patients with schizophrenia are also more prone to seizures. Patients with mental disorders, especially schizophrenia, are often afflicted by diabetes. Glucose homeostasis is altered upon the onset of schizophrenia, indicating that patients are at increased risk of diabetes^30^.

This study identified three further risk variants associated with FPG at nominal p-values of < 8.20E-06. These are rs487321 (recessive, intronic, *CADPS*), rs707927 (additive, intronic in *VARS*, and 2 Kb upstream of *VWA7*), and rs12600570 (additive, intronic, *DHX58*). Of these three suggestive markers, the *CADPS* and [*VARS, VWA7*] markers reached genome-wide significance (p-combined = 1.83E-12 and 3.07E-09, respectively) in meta-analysis that jointly analyzes the data from both the phases.

(i) *CADPS* encodes a calcium-dependent secretion activator involved in the exocytosis of vesicles filled with neurotransmitters and neuropeptides. Interestingly, the activator regulates the recruitment of insulin granules and beta-cell function^31,32^; previous global GWAS associated *CADPS* loci with treatment interaction of sulfonylurea (a glucose-lowering drug) and heart failure-related metabolite levels^21,22^; and GTeX annotates the marker as regulating the expression of its own gene (*CADPS*) in adipose-subcutaneous and tibial artery tissues. Furthermore, as indicated in our results, with a heterozygous genotype at the risk variant, TG was significantly associated with HOMA-IR (p < 0.003) and FPG with HOMA-β (p < 0.003).

(ii) *VARS* encodes valyl-tRNA synthetase and is associated with diabetic cataract, neurodevelopmental disorder, microcephaly, seizures, and cortical atrophy. *VWA7* encodes Von Willebrand Factor A Domain-Containing Protein 7; previous global GWAS associated the *VWA7* locus with IBD, blood plasma proteome, blood protein levels, and schizophrenia. Furthermore, the risk variant and its 26 strong LD partners are from a gene-dense region, commonly referred to as the HLA “class III” region^33^, containing a large number of genes (i.e., *TNF*, *AIF1*, *PRRC2A*, *APOM*, *BAG6*, *C6orf47*, *CSNK2B*, *GPANK1*, *LY6G5B*, *LY6G5C*, *ABHD16A*, *LOC105375018*, *LY6G6F-LY6G6D*, *LY6G6F*, *LY6G6E*, *LY6G6D*, *C6orf25*, *LY6G6C*, *MSH5-SAPCD1*, *MSH5*, *VARS*, *VWA7*, *C6orf48*, *NEU1*, *HSPA1A*, *EHMT2*, and *C2*) (Fig. 2 and Supplementary Table S3). Markers and genes from the HLA region are associated with risk for type 1 diabetes^34^ and type 2 diabetes^35^: *TNF* mediates obesity-related insulin resistance^36^; the *HSPA1A* gene (encoding HSP70) gets upregulated and correlates with HbA1c levels in pregnant women with gestational diabetes^37^; people with type 2 diabetes have higher HSP70 levels in serum correlating with diabetes duration^38^; and an upstream variant of *HSPA1A* (i.e., rs17201192, an LD partner (r^2^ = 0.83) of the reported [*VARS*, *VWA7*] marker) showed an association with FPG, albeit at a nominal p-value of 3.3E-05, in our analysis (see Supplementary Table S3). Our results imply, with genotypes of heterozygosity or homozygosity for the risk allele, significant interactions between FPG and HOMA-β and HOMA-S; and with a heterozygous genotype, interactions between HbA1c and HOMA-IR and HOMA-S. TG was also seen to interact with HOMA-S at p = 0.007 with a heterozygous genotype. The [*VARS*, *VWA7*] variant appeared to regulate the expression of *LY6G5B*, *GPANK1*, *AIF1*, *C6orf25*, *SAPCD1-AS1*, and *TNXA*; previous global GWAS associated these genes with ASD and IBD, which are known to co-occur with type 2 diabetes^39^.

(iii) The *DHX58* gene encodes DExH-box helicase 58. Previous global GWA studies associated a missense variant (i.e., rs2074158-T/*DHX58*), which is in LD (r^2^ = 0.56) with the reported *DHX58* risk variant, with CAD (p-value = 2.0E-10) in UK BioBank populations^17^. We further noticed that the identified ROH region (17:36532212–43490282) (see Table 7) covering the *DHX58* marker overlaps with the ROH (17: 36839131–38938944) (see Table 4 from our previous publication^12^) covering a marker (rs9972882 from *PGAP3*) that is associated with high triglyceride levels^12^.

The presented results indicate that the *DHX58* risk variant regulates *DHX58, RAB5C*, *KCNH4*, and *HSPB9*; interestingly, previous global GWAS implicated these four genes in CAD^17^. Furthermore, markers from *RAB5C* were associated with fibrinogen levels, which are known to be elevated in diabetic patients, especially those with foot ulcers^40^. Our results pointed to significant (p-value < 0.003) interactions between TG and (HOMA-IR and C-peptide levels) and between FPG and HOMA-β at genotypes homozygous for a risk allele.

All the four identified risk variants are intronic; however, as discussed above, genotype-tissue expression data revealed that each of the four variants can regulate genes that are associated with diabetes-related or comorbid disorders. Given that a large burden of homozygosity and excess of recessive alleles are attributed to Arab population from Kuwait^8^, the observations that two of the four identified risk variants appeared when genetic model based on the recessive mode of inheritance was used and that all four variants were in ROH segments are not surprising.

Association tests were examined with both raw and inverse normal transformed FPG values. The reported four associations remained significant when co-variate adjustments were done for diabetes medication, obesity and diagnosis for diabetes. The four associations remained significant when FPG values were preadjusted by a fixed amount per diabetes medication status. Further examination of the identified associations in the sub-cohorts of entirely diabetic patients or of entirely healthy participants revealed that the *RPS6KA1, CADPS* and *VARS* markers performed better in terms of retaining significance in cohorts of diabetic patients and the *DHX58* marker in the cohort of participants free of diabetes.

Consideration of ethnic populations in association studies is supposed to help in enlarging the global catalog of risk loci by way of indicating novel risk loci (not seen in major continental populations). Previous studies from the region on Arab cohorts demonstrated this aspect by way of identifying novel risk loci for type 2 diabetes (T2DM) at either genome-wide significant or suggestive p-values for associations – such loci include *KIF12, DVL1, EPB41L3, DTNB, DLL1, CTNNB1, JAG1, MLXIP, CDKLAL1, TCF7L2, KCTD8, GABRG1, GABRA2, COX7B2, GABRA4, ZNF106* and *OTX2-AS1* (Supplementary Table S5)^41–45^. Our study now adds *RPS6KA1, CADPS*, (*VARS*, *VWA7*), and *DHX58* to this list of novel T2DM risk loci in Arab population.

Because of the nature of the study design that uses HumanOmniExpress BeadChip, the study does not consider genetic variants that are seen only in the Arab population. However, we find that there are statistically significant differences in genotype distributions at the risk variants between the Arab population and continental populations (Supplementary Table S6). The risk allele frequencies also differ substantially across the populations (Supplementary Figure S5). In order to identify Arab-population-specific risk variants (that are not polymorphic in continental population), we need to perform large-scale genome-wide surveys (a combination of GWAS, exome, and genome sequencing and imputation) of the Arab population with diabetes^46^.

**Figure 3:**
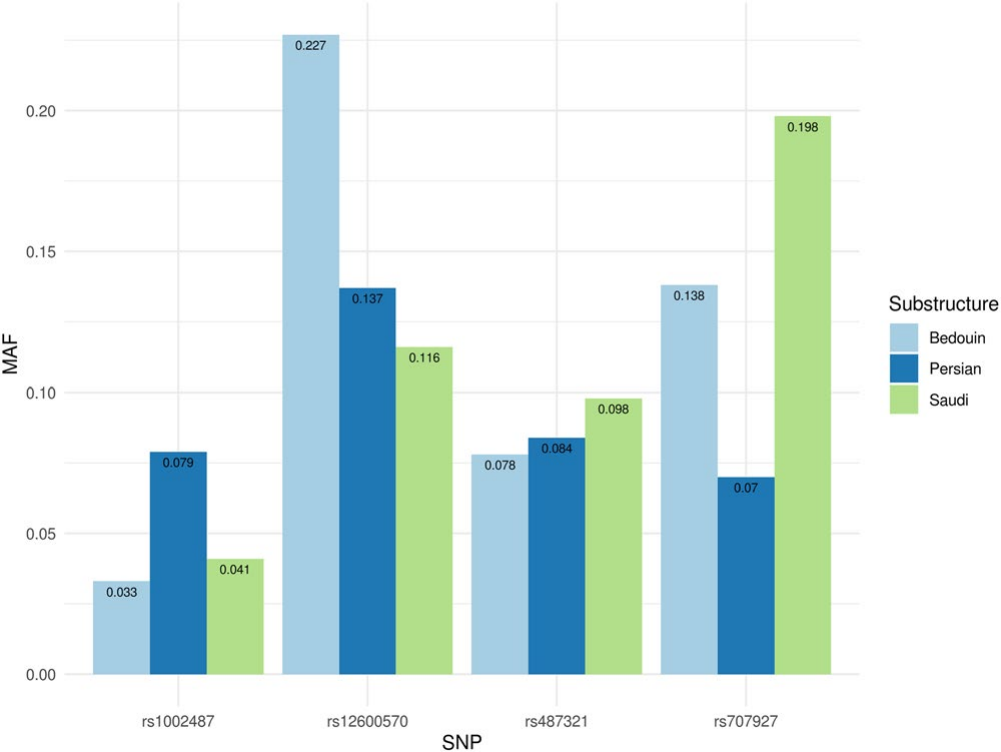
Assessment of allele frequencies at the identified 4 risk variants among the three population substructures of Kuwait. Saudi: Kuwait S subgroup that is predominantly of city-dwelling Saudi Arabian tribe ancestry; Persian: Kuwait P subgroup that is largely of West Asian ancestry, representing Persians; Bedouin: Kuwait B subgroup that is of tent-dwelling Bedouin ancestry^46^.

Our earlier studies identified three population subgroups in Kuwait^8^. the first group (Kuwait P) is largely of West Asian ancestry, representing Persians with European admixture; the second group (Kuwait S) is predominantly of city-dwelling Saudi Arabian tribe ancestry, and the third group (Kuwait B) includes most of the tent-dwelling Bedouin and is characterized by the presence of 17% African ancestry. Allele frequency assessment of the identified 4 risk variants among these substructures (Fig. 3) suggests that the variant rs1002487/*RPS6KA1* is enriched in Persian ancestry, rs12600570/*DHX58* in nomadic Bedouin ancestry, rs707927/*(VARS*, *VWA7)* in Saudi Arabian ancestry followed by nomadic Bedouin ancestry while the frequency of rs487321/*CADPS* is almost equal among the three population substructures of Kuwait.

Limitations of the study include the following: (i) Among study cohorts, there are many subjects assuming hypoglycemic therapy – which we took care by way of adjusting the association tests for medication and by performing sensitivity analysis; however, the such individuals at risk for hyperglycemia might have introduced corrective actions (such as exercise, hypocaloric diet and food supplements) affecting FPG; unfortunately, data relating to these corrective measures were not available and hence we were unable to consider them in association test models or in sensitivity analysis. (ii) The study cohort is relatively small, a limitation which might have hindered the ability to identify more than just the four reported risk variants and to observe any of the established risk variants for glucose-related traits. There is an urgent need to carry out much larger studies on the genetics of diabetes in Arab populations which are notorious for high prevalence of obesity and diabetes^46^.

## Conclusions

This study identified novel risk variants for high FPG in the Arab population of Kuwait. The *RPS6KA1* gene (associated with FPG at genome-wide significance) is known to be involved in glucose homeostasis. Gene loci of *CADPS*, (*VARS*, *VWA7*), and *DHX58* exhibiting nominal associations with FPG were often found to be associated with CAD in previous global GWAS. The identified four associations remained significant when the regression models were adjusted for various confounders (such as medication, obesity and diabetes status) and when the FPG levels were preadjusted by a fixed value per diabetes medication status. The *RPS6KA1, CADPS* and *VARS* markers performed better in terms of retaining significance in cohorts of entirely diabetic patients and the *DHX58* marker in the cohort of participants free of diabetes. With heterozygous or homozygous risk allele genotypes at these risk variants, significant interactions appear to occur between glucose-related and insulin resistance traits. The identified gene loci were previously associated with various other disorders (including IBD, schizophrenia, and autism) that appear to share risk factors with diabetes. This study presents, for the first time, potential associations between the *RPS6KA1* gene loci and high TG, FPG, and HbA1c.

## Methods

### Ethics approval and consent to participate

This study was reviewed and approved by the institutional Ethical Review Committee at Dasman Diabetes Institute, Kuwait. Participant recruitment and blood sample collection were conducted under protocols adopted by the Ethical Review Committee. Signed informed consent was obtained from each participant.

### Study participants

Details on participant recruitment and a description of the study cohorts are presented in our previous paper^12^ (for details, see Supplementary Material: Methods section on Study participants). Briefly, 3,145 participants were recruited from two cohorts in Kuwait. A representative sample of Kuwaiti native adults randomly selected from each of the six governorates of Kuwait formed the first group. Native Kuwaitis visiting our institutional clinics for tertiary medical care or our campaigns formed the second group; such visitors interested in participating were invited later to give blood samples after overnight fasting. We confirmed ethnicity through detailed questioning on parental lineage up to three generations. Data on age, sex, medical history, and medication were also recorded, as were baseline characteristics and vital signs. The discovery cohort was drawn largely from the second group and the replication cohort from the first group. 1,913 of the recruited participants were used for the discovery phase and 1,176 for the replication phase.

### Power calculation

We adopted the “gene only” hypothesis and performed two types of power calculation (for details, see Supplementary Material: Methods section on Power calculation): (Type i): Quanto^47^ was implemented to evaluate sample size and the potential to detect FPG trait variance with 80% power and p-value < 5.0E-08. Marginal genetic effect estimates (R_G_^2^) were made to increment from 0.001 to 0.04 in steps of 0.001 in order to detect genetic effects explaining at least 0.1%–4% of trait variance could be detected. (Type ii): QPowR (https://msu.edu/~steibelj/JP_files/QpowR.html) was used to determine the sample size for achieving 80% power for the study design of two phases (discovery and replication) with total sample size of 2,529, total heritability of 0.05, samples genotyped each of the two phases as ~50% of 2,529, markers typed in the second phase as ~0.2% of the markers typed in the first phase, and type I error rate of 5.0E-08.

### Genotyping in the discovery and replication phases

Genome-wide genotyping was performed on an Illumina HumanOmniExpress Array. Top associating markers in the discovery phase were genotyped in replication phase using TaqMan® SNP Genotyping Assays (Applied Biosystems, Foster City, CA, USA) and ABI 7500 Real-Time PCR System (Applied Biosystems) (for details, see Supplementary Material: Methods section on Sample processing: Discovery phase and Replication phase).

### Quality control analyses

Raw intensity data from all samples were pooled and genotype calling was performed using GenomeStudio software. A series of quality metric thresholds was applied to derive a high-quality set of SNPs and samples (for details, see Supplementary Material: Methods section on Quality control analysis). Samples with a call rate >95% were retained. SNPs with inappropriate call quality were removed. Sex was estimated using GenomeStudio and removed mismatched samples. Strand designations were corrected to the forward strand, and REF/ALT designations were corrected using the design files for HumanOmniExpress BeadChip. Markers with allele frequency (–maf 0.01), and deviation from Hardy–Weinberg equilibrium (HWE <10^−6^) were removed. We derived a set of LD-pruned markers (n = 340,299) by removing markers in LD (r^2^ > 0.5) with others in a sliding window of 50-SNP and the LD-pruned marker set was used to measure relatedness among participants to the extent of third-degree relatives, to perform ancestry estimation (using ADMIXTURE^48^), and principal component analysis (using EIGENSTRAT^49^). One sample per pair of related participants was randomly removed. Samples with abnormal deviations, in the extents of component ancestry elements, from what we had established for the three Kuwaiti population subgroups^8^ were removed as samples of ethnicity mismatch. Outliers in PCA were identified and the corresponding samples were removed.

### Quantitative trait association tests

In discovery phase, all the 632,375 SNPs that passed quality control were used in association tests. Selected markers from discovery phase were tested in the replication phase. Both the additive and recessive genetic models were used in tests for associations with FPG and HbA1c. Two types of corrections were made to the associations tests – “Regular Corrections” involved adjustments for age, sex, and the first 10 principal components; and “Additional Corrections” involved further adjustment for glucose-lowering medication.

### Joint analysis with results from discovery and replication phases

The METAL tool^50^ was used to perform meta-analysis with association test statistics from both the discovery and replication phases. Combined analysis of data from both the phases is believed to enable detecting genetic associations with increased power^51^.

### P-value thresholds to assess significance of associations

Threshold for genome-wide significant p-values were calibrated for the counts of LD-pruned markers (n = 340,299), quantitative traits (n = 2, FPG and HbA1c), genetic models (n = 2, additive or recessive), and correction models for the association tests (n = 2, regular correction and further correction for glucose-lowering medication). The “**stringent**” p-value threshold to keep the type I error rate at 5% got calibrated to 1.84E-08. We further defined a “**nominal**” p-value threshold of (>1.84E-08 and <E-05) to identify “suggestive” associations. P-value threshold for significant associations in replication phase was set at 0.05.

### Identifying runs of homozygosity (ROH)

Runs of Homozygosity (ROH) were identified, using PLINK-1.9, through two approaches: **(Approach-1):** Markers that passed quality control were pruned for LD (r^2^ > 0.9) (n = 568,670) and employed to detect ROH segments using parameters recommended by Howrigan *et al*.^52^ **(Approach-2):** The unpruned marker set (n = 632,375) was employed and parameters deployed by Christofidou *et al*.^53^ were used. Consensus ROH regions were derived for the identified groups of overlapping ROH segments and mean ± SD was calculated (by considering the midpoint of each individual ROH falling in the group). Delineated ROH segments were classified as “known” or “novel” by comparison with ROH signatures discovered in global populations^54^.

### Derivation of insulin resistance traits and association with glucose-related traits

We considered a subset of 283 samples, randomly selected from the replication cohort, and measured C-peptide levels in plasma (for details, see Supplementary Material: Methods section on Derivation of insulin resistance traits). Insulin resistance traits (i.e., HOMA-IR, HOMA-β, and HOMA-S) were calculated using the FPG (mmol/l) and C-peptide (nmol/l) values with the HOMA2 calculator (https://www.dtu.ox.ac.uk/homacalculator/). Multivariate linear regression, corrected for age and sex, was performed to assess interactions between (TG, FPG, HbA1c) and insulin resistance traits with respect to the genotypes at risk variants; standardized beta-coefficients (β_1_) and test significance (p-values) were derived using the R Project for Statistical Computing software (https://www.r-project.org/). The p-value threshold calibrated for multiple testing was 0.003 (=0.05/16); the denominator corresponds to four interaction models on each of the four risk variants.

## Supplementary information

Supplementary Material.
