# Supplementary material for: Genome-wide association study identifies novel risk variants from RPS6KA1, CADPS, VARS, and DHX58 for fasting plasma glucose in Arab population
Source: Sci Rep. 2020 Jan 13;10:152. doi: 10.1038/s41598-019-57072-9 (PMC6957513; doi:10.1038/s41598-019-57072-9)

**SUPPLEMENTARY MATERIAL**

**Genome-wide association study identifies novel risk variants from *RPS6KA1*, *CADPS*, *VARS* and *DHX58* for fasting plasma glucose in Arab population**

Prashantha Hebbar^1,2^, Mohamed Abu-Farha^1,#^, Fadi Alkayal^1,#^, Rasheeba Nizam^1,#^, Naser Elkum^1,3^, Motasem Melhem^1^, Sumi Elsa John^1^, Arshad Channanath^1^, Jehad Abubaker^1^, Abdullah Bennakhi^1^, Ebaa Al-Ozairi^1^, Jaakko Tuomilehto^1,4^, Janne Pitkaniemi^4^, Osama Alsmadi^1,5,@^, Fahd Al-Mulla^1,@^, Thangavel Alphonse Thanaraj^1,@^

^1^Dasman Diabetes Institute, P.O. Box 1180, Dasman 15462, Kuwait.

^2^Doctoral program in Population Health, Faculty of medicine, University of Helsinki, Finland.

^3^Sidra Medical and Research Center, Doha, Qatar.

^4^Department of Public Health, University of Helsinki, Helsinki, Finland.

^5^King Hussein Cancer Center, Amman, Jordan.

^#^, These three authors contributed equally.

**^@^,** Correspondence may be addressed to any of the three authors: e-mail: [oa.12163@khcc.jo](mailto:OA.12163@KHCC.JO) (O.A.); [fahd.almulla@dasmaninstitute.org](mailto:Fahd.almulla@dasmaninstitute.org) (FAM); [alphonse.thangavel@dasmaninstitute.org](mailto:alphonse.thangavel@dasmaninstitute.org); (TAT).

**SUPPLEMENTARY METHODS**

**Study participants**

Recruitment of participants and description of the study cohorts are detailed in our previous study^1^. We recruited 3,145 participants from two cohorts in Kuwait. The first group comprised a random, representative sample of adults of Arab ethnicity from the six governorates of Kuwait. A stratified random sampling technique was used to select participants from the computerized register of the Public Authority of Civil Information. The second group comprised Arab individuals seeking tertiary medical care for diabetes/prediabetes-related disorders at the Dasman Diabetes Institute clinics, visitors to our nutrition programmes and fitness center, visitors to our Open Day Events (that offer various screening services), visitors to our campaigns at primary health centers and blood banks in each of the six governorates of Kuwait; such visitors interested to participate were invited to the institute at a later date to give samples after overnight fasting. Blood samples were collected as per the guidelines of the institutional Ethical Review Committee. Ethnicity was confirmed through detailed questioning on parental lineage up to three generations; data on age, sex, and medical history such as diabetes and cardiovascular diseases (CVD) were recorded. Baseline characteristics and vital signs such as height, weight, waist circumference (WC) and blood pressure were recorded. Details on medications taken by the participants for diabetes and other illnesses were collected. A participant was regarded as affected by T2DM if the diagnosis was known to the participant (self-declaration) or if FPG was ≥7 mmol/L (126 mg/dl) or if HbA1C ≥6.5% (48 mmol/mol). When in doubt, the recorded details on anti-diabetes medication were used; and for participants recruited through our clinics or our campaigns (constituting the above-mentioned second group), the clinician’s notes were used.

Discovery cohort was drawn largely but not exclusively from the second group and replication cohort was drawn largely but not exclusively from the first group; totally, 1,913 people were considered for the discovery phase and 1,176 for the replication phase.

**Sample processing**

A Gentra Puregene® kit (Qiagen, Valencia, CA, USA) was used to extract DNA. Quantification of DNA was performed using Quant-iT™ PicoGreen® dsDNA Assay Kit (Life Technologies, Grand Island, NY, USA) and Epoch Microplate Spectrophotometer; only samples with a ratio in the range of 1.8–2.1 for absorbance at 260 nm to absorbance at 280 nm were used. DNA stocks were then frozen.

**Discovery phase (1,913 participants)**

Illumina HumanOmniExpress array utilizing Infinium® HD Assay Ultra genotyping assay methods were used to perform genome-wide genotyping by following the standard assays. Imaging of the HumanOmniExpress arrays using Illumina iSCAN System. We performed genotyping in batches; 1,097 participants in 20 batches using Illumina HumanOmniExpress-12v1-Multi_H (730,525 markers); 336 participants in five batches using HumanOmniExpress-12v1-1_B (719,665 markers); and 480 participants in six batches using HumanOmniExpress-24v1-0_a (716,503 markers). These three versions of BeadChips differed from one another by at most ~10,000 markers; otherwise the markers were common between the three chip versions.

**Replication phase (1,176** **participants)**

Top markers identified in the discovery phase were selected for replication. TaqMan® SNP Genotyping Assays (Applied Biosystems, Foster City, CA, USA) and ABI 7500 Real-Time PCR System (Applied Biosystems) were used to perform candidate SNP genotyping. Each PCR sample contained 10 ng of genomic DNA, 5× FIREPol® Master Mix (Solis BioDyne, Tartu, Estonia), and 1 µl of 20× TaqMan® SNP Genotyping Assay (Applied Biosystems). The thermal cycling conditions were 60°C for 1 min, 95°C for 15 min, and then, 40 cycles of 95°C for 15 s and 60°C for 1 min. Genotypes ascribed by real-time PCR were validated by direct Sanger sequencing of the PCR products for selected cases of homozygotes and heterozygotes. Sequencing was performed using the BigDye™ Terminator v3.1 Cycle Sequencing FS Ready Reaction Kit (Applied Biosystems), according to the manufacturer’s recommendations, on an Applied Biosystems 3730xl DNA Analyzer (Applied Biosystems).

**Quality control analysis**

We pooled raw intensity data from all samples from all batches, and performed genotype calling using the GenCall algorithm provided in the GenomeStudio software. We applied a series of quality metric thresholds to derive a high-quality set of SNPs and samples: Samples with call rate >95% were selected. SNPs with call rates of <98%, low intensities (AB R Mean ≤ 0.25), poor cluster separation (Cluster Sep < 0.3), heterozygote clusters too close to homozygotes (AB T Mean ≤ 0.2 or ≥0.8), excess heterozygotes (Het Excess ≥ 0.2), and fewer than expected heterozygotes (Het Excess ≤ −0.3) were removed. Sex estimations were performed using GenomeStudio software, and samples with sex mismatches were removed. Duplicated samples were also removed. Strand designations were corrected to the forward strand and REF/ALT designations were corrected using the design files for HumanOmniExpress Bead Chip (available from Illumina). In addition, poor markers with respect to missingness per individual (--mind 0.1), allele frequency (--maf 0.01), missingness per marker (--geno 0.1), and Hardy–Weinberg equilibrium (HWE < 10^−6^) were removed using PLINK^2^. We calculated relatedness among the participants using the “–genome” feature in PLINK with PI_HAT > 0.125 (i.e., third-degree relatives), and one sample per pair of related participants was randomly removed. Our earlier work^3^ demonstrated that the Arab population in Kuwait is characterized by an admixture of six ancestral components that arise from the Human Genome Diversity Project (HGDP) populations of Negev Bedouin, Yoruba, Brahui tribe, Druze, Kalash tribe, and French Basque; the cited work had estimated the extent of each of the six ancestry components in each of the three genetic substructures of the Kuwaiti population. We performed ancestry estimation using ADMIXTURE^4^, through which samples with abnormal deviations in the extents of component ancestry elements were identified as samples of ethnicity mismatch and such samples were removed. These series of quality control steps reduced the number of markers to 632,375 and the size of the discovery cohort to 1,353 samples.

We performed Principal components analysis (PCA) using EIGENSTRAT^5^ with the following parameters: number of eigenvectors to output (numoutevec = 10), no outlier removal (numoutlieriter = 0), number of principal components along which to remove outliers during each outlier removal iteration (numoutlierevec = 10), and number of SDs a participant must exceed, along one of the top (numoutlierevec) principal components, in order for that participant to be classified as an outlier (outliersigmathresh = 6.0) and to be removed. We used all the 10 principal components as covariates to correct the association tests for population stratification.

LD-pruning was performed using ‘–indep-pairwise’ option in PLINK to remove markers with an R^2^ value of >0.5 with any other SNP within a 50-SNP sliding window (advanced in 5-SNP increments). These pruned markers (340,299) were used for measuring relatedness and admixture, to perform PCA and to calibrate genome-wide p-value thresholds used to identify significant genotype-phenotype associations.

**Quantitative trait association tests in discovery phase**

We performed association tests with all the 632,375 SNPs (that passed the QC without LD-pruning) against FPG and HbA1c using linear regression methods under additive and recessive genetic models. Association tests were adjusted for age, sex, and first 10 principal components with further adjustment towards medication for diabetes.

**Power calculation**

We performed two types of power calculations: one to estimate the sample size and its potential to detect variability in quantitative traits with 80% power and a p-value threshold of 5.0E-08; the second one to determine the number of samples required to achieve 80% power in a two-stage (discovery and replication) design.

Quanto software (<http://biostats.usc.edu/Quanto.html>)^6^ was used to perform the first calculation for the consideration of both the additive and recessive models. “Gene only” hypothesis was used. We set the power for the analysis at 80% and considered the type 1 error at a p-value of 5.0E-08 as significant. We set the marginal genetic effect estimate (R_G_^2^) to range from 0.001 to 0.04 in increments of 0.001 so that the genetic effect that explains at least 0.1%–4% of trait variance could be detected. For each of the FPG traits, the population (mean ± standard deviation) of the quantitative trait was used. Upon using the Quanto to estimate the power of our study over a range of % variance of the trait, it was found that the discovery cohort size had 80% power to detect associations with genetic variants (under additive or recessive models) that explained 0.6% variance of the trait. The sample sizes required to detect various R^2^_G_ values in discovery phase were denoted as (R^2^_G_, sample size): (0.001,7845), (0.002,3921), (0.003, 2612), (0.004,1958), (0.005, 1566), (0.006, 1304), (0.007, 1117). The acceptable effect sizes, for associations between the trait of FPG and SNP markers in discovery phase, at different allele frequencies are presented in **Table** **S1.**

For the second calculation, QPowR software (https://msu.edu/~steibelj/JP_files/QpowR.html) was used with the following parameters: total sample size = 2,529; total heritability = 0.05; samples genotyped in the first or second stage = approximately 50% of 2,529; markers typed in the second stage = typically 0.2% of the markers typed in the first stage; and type I error rate = 5.0E-08.

**Derivation of insulin resistance traits**

We considered a subset of 283 samples randomly selected from the replication cohort and measured C-peptide levels in plasma. Blood was drawn into EDTA tubes and the plasma was obtained after centrifugation, aliquoted and then stored at -80°C to be assayed. C-peptide level was evaluated using Ultrasensitive C-peptide ELISA kit from Mercodia (Mercodia, Uppsala, Sweden) according to the manufacturer’s instructions as previously reported^7^. Inter- and intra-assay coefficient of variation was <5%.

**References**

1 Hebbar, P. *et al.* Genome-wide association study identifies novel recessive genetic variants for high TGs in an Arab population. *J Lipid Res*, doi:10.1194/jlr.P080218 (2018).

2 Purcell, S. *et al.* PLINK: a tool set for whole-genome association and population-based linkage analyses. *Am J Hum Genet* **81**, 559-575, doi:10.1086/519795 (2007).

3 Alsmadi, O. *et al.* Genetic substructure of Kuwaiti population reveals migration history. *PLoS One* **8**, e74913, doi:10.1371/journal.pone.0074913 (2013).

4 Alexander, D. H., Novembre, J. & Lange, K. Fast model-based estimation of ancestry in unrelated individuals. *Genome Res* **19**, 1655-1664, doi:10.1101/gr.094052.109 (2009).

5 Price, A. L. *et al.* Principal components analysis corrects for stratification in genome-wide association studies. *Nat Genet* **38**, 904-909, doi:10.1038/ng1847 (2006).

6 Gauderman, W. J. Sample size requirements for association studies of gene-gene interaction. *Am J Epidemiol* **155**, 478-484 (2002).

7 Abu-Farha, M. *et al.* Lack of associations between betatrophin/ANGPTL8 level and C-peptide in type 2 diabetic subjects. *Cardiovascular diabetology* **14**, 112, doi:10.1186/s12933-015-0277-1 (2015).

**LIST OF** **SUPPLEMENTARY TABLES**

**Table S1. Acceptable effect sizes at different allele frequencies for associations with FPG in discovery phase (n=1304).**

**Table S2. Results of statistical association tests for FPG in discovery, replication, and meta-analysis phases when modeled for the recessive and additive modes of inheritance.** Listed are all the associations with p-values<1.0E-05 in discovery phase.

**Table S3. Results of association tests with markers that are in LD (as seen in the regional plots) with the reported top 4 markers (rs1002487, rs487321, rs707927, rs12600570)** that are **associated with FPG.**

**Table S4.** **Results from tests of associations between the risk variants and insulin resistance traits (and TG, FPG and HbA1c).** A sample set of 283 individuals was considered for this analysis.

**Table S5. Top SNPs from previous T2D association studies in Arabs.**

**Table S6. Comparison of genotype distributions at the identified risk variants between the Arab population and continental populations.**

**LIST OF SUPPLEMENTARY FIGURES**

**Figure S1. Scatter plots representing the first three principal components of the discovery cohort and representative HGDP populations.** The dynamic view of the plot is available at <http://dgr.dasmaninstitute.org/tcn2_pca/index.html>. The reference populations include: West Asia: Negev Bedouin (from Israel, denoting Arabian ancestry), Druze (from Israel), Palestinian (from central region of Israel); Central and South Asia: Brahui tribe (Balochistan in Pakistan); Africa: Mozabite (from North Africa); Europe: French Basque. This figure is as reproduced from our previous publication of [Hebbar et al (2017) The TCN2 variant of rs9606756 [Ile23Val] acts as risk loci for obesity-related traits and mediates by interacting with Apo-A1. *Obesity (Silver Spring)* **25**: 1098-1108].

**Figure S2. Published PCA plot for native Kuwaitis of Arab ethnicity confirmed through detailed surname lineage analysis.** This figure is as reproduced from our previous publication of [Alsmadi et al. (2013) Genetic substructure of Kuwaiti population reveals migration history. *PLoS One* **8**: e74913].

**Figure S3. Intensity maps** **for the 22 markers (selected for replication phase).** The plots depict the observed genotype clusters. The three highlighted clusters in each of the plots display the quality of the three different genotypes called - homozygous allele A (red), heterozygous AB (purple) and homozygous allele B (blue) – in the three genotyping arrays used to genotype the discovery cohort. The 22 markers are: rs1002487; rs4143782; rs12488539; rs6762914; rs487321; rs17065898; rs707927; rs1145784; rs2522219; rs1199028; rs2599723; rs3812689; rs918988; rs1151501; rs11179003; rs7329697; rs4646213; rs3784240; rs1256826; rs930514; rs12600570; rs9959376.

**Figure S4. Manhattan plots of the 632,375 SNPs employed to interrogate the genomes of the study population for associations with the FPG trait, under recessive or additive mode of inheritance.** Labelling is done by considering the threshold of P<1.84E-08 for genome wide significance.

**Figure S5. Comparison of risk allele frequencies at the identified risk variants across populations.**

**SUPPLEMENTARY TABLES**

**Table S1. Acceptable effect sizes at different allele frequencies for associations with FPG in discovery phase (n=1304).**

| **Allele Frequency** | **R-Square (Marginal effect)** | **Power (%)** | **Sample Size** | **Acceptable Effect Size for the FPG trait with Additive model.** | **Acceptable Effect Size for the FPG trait with Recessive model.** |
| --- | --- | --- | --- | --- | --- |
| 0.05 | 0.006 | 80 | 1304 | 0.8972 | 5.5375 |
| 0.06 | 0.006 | 80 | 1304 | 0.8234 | 4.6172 |
| 0.07 | 0.006 | 80 | 1304 | 0.7664 | 3.9602 |
| 0.08 | 0.006 | 80 | 1304 | 0.7208 | 3.4678 |
| 0.09 | 0.006 | 80 | 1304 | 0.6833 | 3.0851 |
| 0.1 | 0.006 | 80 | 1304 | 0.6518 | 2.7792 |
| 0.11 | 0.006 | 80 | 1304 | 0.6249 | 2.5293 |
| 0.12 | 0.006 | 80 | 1304 | 0.6017 | 2.3212 |
| 0.13 | 0.006 | 80 | 1304 | 0.5814 | 2.1454 |
| 0.14 | 0.006 | 80 | 1304 | 0.5635 | 1.9949 |
| 0.15 | 0.006 | 80 | 1304 | 0.5476 | 1.8646 |
| 0.16 | 0.006 | 80 | 1304 | 0.5334 | 1.7509 |
| 0.17 | 0.006 | 80 | 1304 | 0.5206 | 1.6507 |
| 0.18 | 0.006 | 80 | 1304 | 0.509 | 1.5618 |
| 0.19 | 0.006 | 80 | 1304 | 0.4984 | 1.4824 |
| 0.2 | 0.006 | 80 | 1304 | 0.4888 | 1.4112 |
| 0.21 | 0.006 | 80 | 1304 | 0.4801 | 1.3468 |
| 0.22 | 0.006 | 80 | 1304 | 0.472 | 1.2885 |
| 0.23 | 0.006 | 80 | 1304 | 0.4646 | 1.2354 |
| 0.24 | 0.006 | 80 | 1304 | 0.4578 | 1.1869 |
| 0.25 | 0.006 | 80 | 1304 | 0.4516 | 1.1424 |
| 0.26 | 0.006 | 80 | 1304 | 0.4458 | 1.1015 |
| 0.27 | 0.006 | 80 | 1304 | 0.4404 | 1.0637 |
| 0.28 | 0.006 | 80 | 1304 | 0.4355 | 1.0288 |
| 0.29 | 0.006 | 80 | 1304 | 0.4309 | 0.9964 |
| 0.3 | 0.006 | 80 | 1304 | 0.4267 | 0.9663 |
| 0.31 | 0.006 | 80 | 1304 | 0.4228 | 0.9383 |
| 0.32 | 0.006 | 80 | 1304 | 0.4192 | 0.9121 |
| 0.33 | 0.006 | 80 | 1304 | 0.4158 | 0.8877 |
| 0.34 | 0.006 | 80 | 1304 | 0.4128 | 0.8648 |
| 0.35 | 0.006 | 80 | 1304 | 0.41 | 0.8434 |
| 0.36 | 0.006 | 80 | 1304 | 0.4074 | 0.8233 |
| 0.37 | 0.006 | 80 | 1304 | 0.405 | 0.8045 |
| 0.38 | 0.006 | 80 | 1304 | 0.4028 | 0.7867 |
| 0.39 | 0.006 | 80 | 1304 | 0.4009 | 0.77 |
| 0.4 | 0.006 | 80 | 1304 | 0.3991 | 0.7543 |
| 0.41 | 0.006 | 80 | 1304 | 0.3976 | 0.7395 |
| 0.42 | 0.006 | 80 | 1304 | 0.3962 | 0.7255 |
| 0.43 | 0.006 | 80 | 1304 | 0.395 | 0.7123 |
| 0.44 | 0.006 | 80 | 1304 | 0.3939 | 0.6999 |
| 0.45 | 0.006 | 80 | 1304 | 0.393 | 0.6881 |
| 0.46 | 0.006 | 80 | 1304 | 0.3923 | 0.677 |
| 0.47 | 0.006 | 80 | 1304 | 0.3918 | 0.6666 |
| 0.48 | 0.006 | 80 | 1304 | 0.3914 | 0.6567 |
| 0.49 | 0.006 | 80 | 1304 | 0.3912 | 0.6474 |

**Table S2. Results of statistical association tests for FPG in discovery, replication, and meta-analysis phases when modeled for the recessive and additive modes of inheritance.** Listed are all the associations with p-values<1.0E-05 in discovery phase.

| **SNP: Effect Allele: Trait** | **Gene: functional consequences** | **Phase** | **Effect Size^R^** | **P-value^R^** | **Effect Size^DM^** | **P-value^DM^** |
| --- | --- | --- | --- | --- | --- | --- |
| rs1002487: C^#^, FPG | *RPS6KA1*: intronic | Discovery | 8.315 | 1.64E-08 | 8.297 | 1.58E-08 |
|  |  | Replication | 3.442 | 3.7E-04 | 3.509 | 2.15E-04 |
|  |  | Meta | 6.551 | 5.72E-11 | 6.652 | 2.89E-11 |
|  |  |  |  |  |  |  |
| rs4143782: T^@^, FPG | *HS6ST1*: intronic | Discovery | 0.8754 | 1.35E-07 | 0.8784 | 1.14E-07 |
|  |  | Replication | 0.1207 | 0.5617 | 0.0409 | 0.7403 |
|  |  | Meta | 4.0868 | 4.739E-05 | 3.910 | 9.21E-05 |
|  |  |  |  |  |  |  |
| rs487321: A^#^, FPG | *CADPS*: intronic | Discovery | 6.133 | 1.53E-07 | 6.161 | 1.23E-07 |
|  |  | Replication | 3.955 | 2.25E-06 | 3.88 | 3.033E-06 |
|  |  | Meta | 7.047 | 1.83E-12 | 7.031 | 2.054E-12 |
|  |  |  |  |  |  |  |
| rs707927: G^@^, FPG | *VARS*, *VWA7*: intron in *VARS*, 2 Kb upstream of *VWA7* | Discovery | 0.9453 | 8.24E-06 | 0.9262 | 1.19E-05 |
|  |  | Replication | 0.6375 | 8.25E-05 | 0.6503 | 3.18E-05 |
|  |  | Meta | 5.928 | 3.074E-09 | 6.033 | 1.61E-09 |
|  |  |  |  |  |  |  |
| rs1145784: G^#^, FPG | *LOC107986623*: intronic | Discovery | 5.824 | 2.02E-08 | 5.778 | 2.38E-08 |
|  |  | Replication | 0.1172 | 0.8294 | 0.2319 | 0.6638 |
|  |  | Meta | 4.234 | 2.29E-05 | 4.363 | 1.28E-05 |
|  |  |  |  |  |  |  |
| rs2522219: A^#^, FPG | *DOCK4*: intronic | Discovery | 5.623 | 2.33E-08 | 9.278 | 1.55E-08 |
|  |  | Replication | NA | NA | NA | NA |
|  |  | Meta | 5.585 | 2.33E-08 | 5.655 | 1.56E-08 |
|  |  |  |  |  |  |  |
| rs2599723: G^#^, FPG | *NDUFAF6*: intronic | Discovery | 8.65 | 1.63E-07 | 8.62 | 1.65E-07 |
|  |  | Replication | 1.522 | 0.0873 | 1.533 | 0.0831 |
|  |  | Meta | 4.858 | 1.18E-06 | 4.873 | 1.099E-06 |
|  |  |  |  |  |  |  |
| rs3812689: A^#^, FPG | *HK1*: intronic | Discovery | 1.259 | 3.64E-08 | 5.915 | 7.44E-08 |
|  |  | Replication | -0.994 | 0.2608 | -0.8908 | 0.3041 |
|  |  | Meta | 3.242 | 1.18E-03 | 3.216 | 1.3E-03 |
|  |  |  |  |  |  |  |
| rs1151501: A^@^, FPG | *KAT2*, *RNASEH2C*: downstream 500B, intronic | Discovery | 0.926 | 5.68E-06 | 0.9465 | 3.31E-06 |
|  |  | Replication | -0.0502 | 0.8251 | -0.05259 | 0.741 |
|  |  | Meta | 2.983 | 2.85E-03 | 2.982 | 2.86E-03 |
|  |  |  |  |  |  |  |
| rs11179003: T^#^, FPG | *TPH2*: intronic | Discovery | 6.087 | 3.205E-08 | 6.071 | 3.166E-08 |
|  |  | Replication | 2.194 | 0.1304 | 2.325 | 0.1067 |
|  |  | Meta | 4.920 | 8.637E-07 | 4.995 | 5.898E-07 |
|  |  |  |  |  |  |  |
| rs4646213: A^#^, FPG | *SLC15A1*: intronic | Discovery | 6.006 | 1.00E-08 | 5.893 | 1.76E-08 |
|  |  | Replication | 0.1351 | 0.8367 | 0.2238 | 0.7275 |
|  |  | Meta | 4.316 | 1.59E-05 | 4.343 | 1.41E-05 |
|  |  |  |  |  |  |  |
| rs3784240: A^#^, FPG | *JAG2*: intronic | Discovery | 6.372 | 7.84E-09 | 6.4 | 6.06E-09 |
|  |  | Replication | 1.562 | 0.0778 | 1.246 | 0.152 |
|  |  | Meta | 5.415 | 6.13E-08 | 5.220 | 1.78E-07 |
|  |  |  |  |  |  |  |
| rs1256826: A^@^, FPG | *ARPIN*, *C15orf38*-*AP3S2*: intronic | Discovery | 1.013 | 5.36E-07 | 0.9968 | 7.68E-07 |
|  |  | Replication | 0.2108 | 0.6943 | 0.2877 | 0.5891 |
|  |  | Meta | 3.755 | 1.73E-04 | 3.813 | 1.37E-04 |
|  |  |  |  |  |  |  |
| rs930514: G^@^, FPG | *PITPNM3*, *KIAA0753*: intergenic | Discovery | 0.5489 | 2.22E-05 | 0.5222 | 5.18E-05 |
|  |  | Replication | 0.1999 | 0.1593 | 0.1184 | 0.2019 |
|  |  | Meta | 3.951 | 7.77E-05 | 3.946 | 7.94E-05 |
|  |  |  |  |  |  |  |
| rs12600570: T^@^, FPG | *DHX58*: intronic | Discovery | 0.8166 | 7.49E-06 | 0.8374 | 4.11E-06 |
|  |  | Replication | 0.3892 | 4.67E-03 | 0.3682 | 5.65E-03 |
|  |  | Meta | 5.142 | 2.715E-07 | 5.186 | 2.15E-07 |
|  |  |  |  |  |  |  |
| rs9959376: T^#^, FPG | *LOC105371956*, *LOC105371957*: intronic | Discovery | -2.607 | 5.91E-04 | -2.669 | 4.2E-04 |
|  |  | Replication | -0.7977 | 0.1385 | -0.9115 | 0.0882 |
|  |  | Meta | -3.448 | 5.64E-04 | -3.672 | 2.41E-04 |
| rs17065898: C^@^, FPG | *LOC105377703*: intronic | Discovery | 0.8277 | 3.09E-07 | 0.8006 | 7.29E-07 |
|  |  | Replication | 0.4964 | 0.1093 | 0.5557 | 0.07107 |
|  |  | Meta | 4.694 | 2.683E-06 | 4.727 | 2.274E-06 |
|  |  |  |  |  |  |  |
| rs918988: C^@^, FPG | *NAV2*: intronic | Discovery | 0.5951 | 3.19E-06 | 0.5969 | 2.77E-06 |
|  |  | Replication | 0.5696 | 0.2965 | -0.09201 | 0.3445 |
|  |  | Meta | 3.979 | 6.93E-05 | 2.563 | 1.04E-02 |
|  |  |  |  |  |  |  |
| **The following markers failed SNP quality assessment tests (see Table 2)** | | | | | | |
| rs12488539: T^@^, FPG | *ARF4*-*AS1*, *PDE12*: downstream 500B, intronic | Discovery | -0.5788 | 5.19E-05 | -0.5753 | 5.48E-05 |
|  |  | Replication | -0.6336 | 0.07939 | -0.0615 | 0.0861 |
|  |  | Meta | -4.066 | 4.78E-05 | -4.029 | 5.59E-05 |
|  |  |  |  |  |  |  |
| rs6762914: C^@^, FPG | *DENND6A*, *PDE12*: intronic | Discovery | -0.5246 | 5.15E-04 | -0.5297 | 1.29E-04 |
|  |  | Replication | -0.6303 | 0.1178 | -0.6124 | 0.1259 |
|  |  | Meta | -3.746 | 1.80E-04 | -3.755 | 1.73E-04 |
|  |  |  |  |  |  |  |
| rs1199028: C^#^, FPG | *ZNF704*: intronic | Discovery | 3.153 | 9.67E-07 | 3.177 | 7.23E-07 |
|  |  | Replication | -0.6192 | 0.03386 | -0.5468 | 0.05376 |
|  |  | Meta | 2.474 | 0.0133 | 2.639 | 8.31E-03 |
|  |  |  |  |  |  |  |
| rs7329697: C^#^, FPG | *LINC00540*: intronic | Discovery | 5.718 | 4.09E-10 | 5.76 | 2.687E-10 |
|  |  | Replication | 0.1551 | 0.6921 | 0.2868 | 0.4523 |
|  |  | Meta | 5.102 | 3.36E-07 | 5.377 | 7.58E-08 |
|  |  |  |  |  |  |  |

**^EffectSize^**, Effect size represents beta value for discovery and replication phases, and Z-score for meta-analysis. R-regular correction: Corrected for age, sex and the top 10 principal components that resulted from the Principal Components Analysis of the genotype data; DM: Corrected for diabetes medication in addition to the regular correction.

**^#^**, association with the trait was observed under the genetic model based on recessive mode of inheritance; **^@^**, association with the trait was observed under the genetic model based on additive mode of inheritance.

**Table S3.** **Results of association tests with markers that are in LD (as seen in the regional plots) with the reported top 4 markers (rs1002487, rs487321, rs707927, rs12600570)** that are **associated with FPG.**

| **SNP** | **Chromosomal Position** | **LD(r^2^≥0.2)** | **Reference/ Alternate allele** | **Effect allele and Frequency** | **Gene: variant type** | **Effect size** | **P-value** |
| --- | --- | --- | --- | --- | --- | --- | --- |
| 1. **rs1002487 (*RPS6KA1*)** | | | | | | | |
| **rs1002487** | **1:26865971** | **1** | **T/C** | **C^#^: 0.0595** | ***RPS6KA1*: intronic** | **8.315** | **1.64E-08** |
| rs6668958 | 1:26902388 | 0.238923 | T/G | G^#^: 0.1494 | *RPS6KA1*: upstream | 1.382 | 2.89E-02 |
| 1. **rs487321 (***CADPS*) | | | | | | | |
| **rs487321** | **3:62790623** | **1** | **A/G** | **A^#^: 0.0821** | ***CADPS*: intronic** | **6.133** | **1.529E-07** |
| rs562511 | 3:62779104 | 0.33926 | G/A | G^#^: 0.049 | *CADPS*: intronic | 0.8203 | 0.5479 |
| rs1513135 | 3:62803217 | 0.228568 | T/G | T^#^: 0.0594 | *CADPS*: intronic | 2.494 | 0.2835 |
| 1. **rs707927** (*VARS*, *VWA7*) | | | | | | | |
| **rs707927** | **6:31745518** | **1** | **A/G** | **G^@^: 0.1062** | ***VARS*, *VWA7*: intronic variant, upstream variant 2KB** | **0.9453** | **8.24E-06** |
|  |  |  |  |  |  |  |  |
| rs9468857 | 6:30963143 | 0.249472 | A/G | G^@^: 0.0653 | *MUC21*, *MUC22*: intergenic | 1.072 | 2.86E-05 |
| rs2596574 | 6:31334174 | 0.201713 | G/A | A^@^: 0.1506 | *DHFRP2*: exonic | 0.3919 | 2.59E-02 |
| rs2844556 | 6:31341357 | 0.207191 | G/A | A^@^: 0.1487 | *RNU6*-*283P*, *FGFR3P1*: intergenic | 0.3729 | 3.71E-02 |
| rs2507980 | 6:31347877 | 0.215266 | G/A | A^@^: 0.1463 | *FGFR3P1*, *ZDHHC20P2*: intergenic | 0.3839 | 3.54E-02 |
| rs2523483 | 6:31353792 | 0.383029 | T/G | G^@^: 0.0941 | *LOC105379656, LOC105379664*: non-coding transcript variant, upstream variant 2KB | 0.4285 | 4.39E-02 |
| rs2523477 | 6:31360389 | 0.220306 | T/C | C^@^: 0.1466 | *MICA-AS1*: upstream | 0.3791 | 3.79E-02 |
| rs2523476 | 6:31361424 | 0.218079 | C/T | T^@^: 0.146 | *MICA-AS1*: upstream | 0.3838 | 3.51E-02 |
| rs2523464 | 6:31363489 | 0.215556 | G/A | A^@^: 0.1429 | *LOC101929072*, MICA-AS1: intronic | 0.3964 | 3.18E-02 |
| rs28366135 | 6:31364105 | 0.203146 | A/G | G^@^: 0.153 | LOC101929072, MICA-AS1 | 0.3674 | 3.75E-02 |
| rs2844523 | 6:31368588 | 0.217063 | G/A | A^@^: 0.1458 | LOC101929072, MICA-AS1, MICA: intronic variant, upstream variant 2KB | 0.3772 | 3.85E-02 |
| rs2263313 | 6:31373810 | 0.216216 | G/A | A^@^: 0.1453 | MICA: intronic | 0.3908 | 3.26E-02 |
| rs2857281 | 6:31374262 | 0.216589 | A/C | C^@^: 0.1478 | MICA: intronic | 0.3223 | 7.65E-02 |
| rs16899646 | 6:31416920 | 0.218813 | C/G | G^@^: 0.2201 | LINC01149, HLA-X: intergenic | -0.0492 | 0.7467 |
| rs6920723 | 6:31421489 | 0.439785 | G/A | A^@^: 0.0775 | LINC01149, HLA-X: intergenic | 0.7304 | 2.67E-03 |
| rs2255001 | 6:31430143 | 0.246056 | G/C | C^@^: 0.1886 | HCP5: upstream 2KB | 0.2513 | 0.1271 |
| rs2243621 | 6:31431820 | 0.241543 | C/T | T^@^: 0.1899 | HCP5: non-coding transcript variant | 0.247 | 0.134 |
| rs2516482 | 6:31496569 | 0.317821 | A/G | G^@^: 0.1143 | MCCD1: upstream variant 2KB | 0.3233 | 0.1081 |
| rs1800629 | 6:31543031 | 0.328123 | G/A | A^@^: 0.1162 | TNF: upstream variant 2KB | 0.2928 | 0.1368 |
| rs2736182 | 6:31583312 | 0.659117 | G/A | A^@^: 0.1302 | AIF1: missense, upstream variant 2KB, 5’ untranslated region | 0.7111 | 2.58E-04 |
| rs2736163 | 6:31597708 | 0.689049 | C/T | T^@^: 0.1265 | PRRC2A: intronic | 0.6628 | 8.62E-04 |
| rs2736161 | 6:31598293 | 0.833381 | G/A | A^@^: 0.109 | PRRC2A: intronic | 0.847 | 5.66E-05 |
| rs1046089 | 6:31602967 | 0.225266 | G/A | A^@^: 0.3012 | PRRC2A: missense | 0.2446 | 8.53E-02 |
| rs2242656 | 6:31614102 | 0.238735 | C/T | C^@^: 0.3031 | BAG6: intronic | 0.1444 | 0.3042 |
| rs2844463 | 6:31615167 | 0.228788 | G/A | A^@^: 0.3126 | BAG6: intronic | 0.1155 | 0.4114 |
| rs805300 | 6:31618567 | 0.69563 | A/G | G^@^: 0.126 | APOM, BAG6: intronic variant, upstream variant 2KB | 0.6547 | 1.06E-03 |
| rs805264 | 6:31623873 | 0.829622 | G/A | A^@^: 0.1064 | APOM, BAG6: intronic | 0.8303 | 8.07E-05 |
| rs707921 | 6:31625541 | 0.691521 | C/A | A^@^: 0.1261 | APOM, BAG6: intronic | 0.6355 | 1.41E-03 |
| rs805263 | 6:31628113 | 0.903759 | A/C | C^@^: 0.1007 | BAG6, C6orf47: UTR variant 5 prime | 0.8937 | 3.94E-05 |
| rs805259 | 6:31631377 | 0.691575 | A/G | G^@^: 0.126 | GPANK1: intronic | 0.6365 | 1.38E-03 |
| rs805258 | 6:31633552 | 0.829534 | C/T | T^@^: 0.1089 | CSNK2B, GPANK1: intronic variant, upstream variant 2KB, 5’ untranslated region | 0.837 | 6.89E-05 |
| rs14365 | 6:31635710 | 0.259248 | T/C | C^@^: 0.2619 | CSNK2B, GPANK1: synonymous codon, upstream variant 2KB | 0.1415 | 0.3398 |
| rs805268 | 6:31638178 | 0.833381 | A/C | C^@^: 0.109 | CSNK2B, LY6G5B: downstream variant 500B, upstream variant 2KB | 0.847 | 5.66E-05 |
| rs805267 | 6:31639757 | 0.6952 | G/A | A^@^: 0.1264 | LY6G5B: missense | 0.6519 | 1.04E-03 |
| rs805272 | 6:31641386 | 0.833381 | G/A | T^@^: 0.2125 | LY6G5B, LY6G5C: intergenic | 0.847 | 5.66E-05 |
| rs805269 | 6:31644282 | 0.6952 | A/G | G^@^: 0.1264 | LY6G5C: downstream variant 500B | 0.6519 | 1.04E-03 |
| rs805283 | 6:31657924 | 0.694881 | G/A | A^@^: 0.1269 | ABHD16A, LOC105375018: intronic | 0.6573 | 9.53E-04 |
| rs805273 | 6:31665452 | 0.368752 | G/A | A^@^: 0.2125 | ABHD16A: intronic | 0.2174 | 0.171 |
| rs1266074 | 6:31666551 | 0.387564 | C/T | T^@^: 0.2142 | ABHD16A: intronic | 0.285 | 0.2383 |
| rs805295 | 6:31675297 | 0.685379 | C/T | T^@^: 0.2142 | LY6G6F-LY6G6D, LY6G6F: missense | 0.6199 | 1.76E-03 |
| rs9469040 | 6:31679874 | 0.587469 | G/T | T^@^: 0.1163 | LY6G6E, LY6G6F-LY6G6D: downstream variant 500B, intronic variant, non-coding transcript variant | 0.539 | 8.66E-03 |
| rs9469042 | 6:31683018 | 0.523164 | T/C | C^@^: 0.1304 | LY6G6D, LY6G6E, LY6G6F-LY6G6D: intronic variant, upstream variant 2KB | 0.4327 | 2.63E-02 |
| rs3749952 | 6:31683157 | 0.589902 | T/G | G^@^: 0.1175 | LY6G6D, LY6G6E, LY6G6F-LY6G6D: intronic variant, missense, upstream variant 2KB | 0.5213 | 1.08E-02 |
| rs4713479 | 6:31688799 | 0.589902 | C/T | T^@^: 0.1175 | C6orf25, LY6G6C: intronic | 0.5213 | 1.08E-02 |
| rs9296001 | 6:31722262 | 0.732693 | C/T | T^@^: 0.0968 | MSH5-SAPCD1, MSH5: intronic | 0.7603 | 5.36E-04 |
| rs9461718 | 6:31727822 | 0.736267 | A/C | C^@^: 0.0972 | MSH5-SAPCD1, MSH5: intronic | 0.7786 | 3.85E-04 |
| rs9461721 | 6:31738408 | 0.749992 | A/G | G^@^: 0.0965 | VWA7: intronic | 0.7895 | 3.29E-04 |
| rs11531 | 6:31750509 | 0.816505 | G/A | A^@^: 0.0887 | VARS: missense, noncoding transcript variant | 0.923 | 4.27E-05 |
| rs9469054 | 6:31761063 | 0.849017 | G/A | A^@^: 0.092 | VARS: intronic variant, upstream variant 2KB | 0.9125 | 4.05E-05 |
| rs17201192 | 6:31786365 | 0.83091 | G/T | T^@^: 0.091 | HSPA1A: upstream | 0.9239 | 3.30E-05 |
| rs9469063 | 6:31813576 | 0.834456 | C/G | G^@^: 0.090 | C6orf48, NEU1: intergenic | 0.8953 | 6.07E-05 |
| rs9501159 | 6:31860118 | 0.749519 | G/A | A^@^: 0.0865 | EHMT2: intronic | 0.7824 | 6.85E-04 |
| rs17201403 | 6:31888062 | 0.713578 | G/A | A^@^: 0.0829 | C2: intronic | 0.7947 | 7.45E-04 |
| rs9332704 | 6:31895619 | 0.648678 | G/T | A^@^: 0.0872 | C2: intronic | 0.8147 | 4.12E-04 |
| rs10046127 | 6:32017521 | 0.308016 | C/T | T^@^: 0.158 | TNXB: intronic | 0.3472 | 4.90E-02 |
| rs34878747 | 6:32052898 | 0.314975 | C/T | T^@^: 0.1595 | TNXB: intronic | 0.31 | 8.43E-02 |
| rs204899 | 6:32057627 | 0.232217 | C/T | T^@^: 0.2078 | TNXB: intronic | 0.2302 | 0.145 |
| rs13196724 | 6:32081422 | 0.341973 | C/G | G^@^: 0.1453 | TNXB, ATF6B: intergenic | 0.3365 | 7.12E-02 |
| rs34129552 | 6:32130329 | 0.23102 | C/G | G^@^: 0.1119 | PPT2-EGFL8, PPT2: intronic | 0.07482 | 0.7911 |
| rs34280838 | 6:32139565 | 0.206242 | T/C | C^@^: 0.1338 | AGPAT1, MIR6721: intronic variant, upstream variant 2KB | 0.3159 | 9.67E-02 |
| rs17576984 | 6:32212985 | 0.226403 | C/T | T^@^: 0.1829 | NOTCH4, LOC101929163: intergenic | 0.2521 | 0.1333 |
| rs28366174 | 6:32266490 | 0.268285 | T/C | C^@^: 0.1021 | C6orf10, LOC101929163: intronic | 0.5448 | 1.01E-02 |
| 1. **rs12600570 (***DHX58*) | | | | | | | |
| **rs12600570** | **17:40261545** | **1** | **C/T** | **T^@^: 0.1482** | **DHX58: intronic** | **0.8166** | **7.49E-06** |
| rs2074158 | 17:40257163 | 0.561772 | T/C | C^@^:0.2354 | DHX58:missense, noncoding transcript variant | 0.6122 | 7.112E-05 |
| rs11079033 | 17:40262472 | 0.481461 | A/G | G^@^: 0.2683 | DHX58: intronic | 0.6214 | 2.07E-05 |
| rs4796770 | 17:40264896 | 0.531474 | C/A | A^@^: 0.0902 | DHX58, KAT2A: downstream variant 500B, intronic variant, upstream variant 2KB | 0.8614 | 1.22E-04 |
| rs8072215 | 17:40271970 | 0.321944 | A/G | G^@^: 0.3469 | KAT2A: intronic | 0.4017 | 2.10E-03 |
| rs8081327 | 17:40274200 | 0.365853 | G/A | A^@^: 0.3112 | HSPB9, KAT2A: upstream variant 2KB | 0.3684 | 6.12E-03 |
| rs1122326 | 17:40274873 | 0.367543 | A/C | C^@^: 0.2983 | HSPB9, KAT2A: missense, upstream variant 2KB | 0.2527 | 7.01E-02 |
| rs11079035 | 17:40289012 | 0.492018 | G/A | A^@^: 0.247 | RAB5C: intronic variant, upstream variant 2KB | 0.3935 | 5.53E-03 |
| rs12603327 | 17:40290552 | 0.369501 | T/C | C^@^: 0.3117 | RAB5C: intronic variant | 0.3635 | 7.01E-03 |
|  |  |  |  |  |  |  |  |

**Table S4. Results from tests of associations between the risk variants and insulin resistance traits (and TG, FPG and HbA1c).** A sample set of 283 individuals was considered for this analysis.

| SNP_Allele^model^ | Trait | Beta^R^ | P-value^R^ | Beta^DM^ | P-value^DM^ |
| --- | --- | --- | --- | --- | --- |
| rs1002487_C^Rec^  ***RPS6KA1*** | FPG | 9.512 | 2.092E-06 | 9.507 | 2.23E-06 |
|  | HbA1c | 5.189 | 1.594E-05 | 5.198 | 1.61E-05 |
|  | TG | 145.6 | 3.24E-08 | 145.2 | 3.68E-08 |
|  | C-peptide | -0.2022 | 0.3903 | -0.207 | 0.3796 |
|  | HOMA-IR | 5.832 | 1.047E-14 | 5.83 | 1.21E-14 |
|  | HOMA-β | -56.32 | 0.0626 | -56.41 | 0.0628 |
|  | HOMA-S | -23.05 | 0.5002 | -22.09 | 0.5039 |
| rs707927_G^Add^  ***[VARS, VWA7]*** | FPG | 1.534 | 0.00095 | 1.544 | 0.00098 |
|  | HbA1c | 0.7361 | 0.0082 | 0.7504 | 0.00754 |
|  | TG | 2.03 | 0.7434 | 1.59 | 0.7993 |
|  | C-peptide | -0.024 | 0.6502 | -0.029 | 0.5917 |
|  | HOMA-IR | 0.3495 | 0.0543 | 0.3485 | 0.0571 |
|  | HOMA-β | -16.41 | 0.0177 | -16.68 | 0.0168 |
|  | HOMA-S | 2.302 | 0.769 | 2.491 | 0.7528 |
| rs487321_A^Rec^  ***CADPS*** | FPG | 15.9 | 1.36E-08 | 16.32 | 8.21E-09 |
|  | HbA1c | 5.243 | 0.00214 | 5.322 | 0.00207 |
|  | TG | -10.82 | 0.7766 | -7.564 | 0.844 |
|  | C-peptide | -0.0324 | 0.9221 | -0.0008 | 0.998 |
|  | HOMA-IR | 3.662 | 0.00096 | 3.781 | 0.00075 |
|  | HOMA-β | -94.95 | 0.0256 | -96.64 | 0.0248 |
|  | HOMA-S | -78.01 | 0.1046 | -80.77 | 0.0963 |
| rs12600570_T^Add^  ***DHX58*** | FPG | 0.5284 | 0.1307 | 0.5328 | 0.1285 |
|  | HbA1c | 0.1563 | 0.4551 | 0.1556 | 0.4585 |
|  | TG | -0.282 | 0.9515 | -0.153 | 0.973 |
|  | C-peptide | -0.053 | 0.1849 | -0.052 | 0.195 |
|  | HOMA-IR | -0.053 | 0.697 | -0.0514 | 0.7061 |
|  | HOMA-β | -5.701 | 0.272 | -5.702 | 0.2732 |
|  | HOMA-S | 2.268 | 0.698 | 2.23 | 0.7042 |

**Table S5. Top SNPs from previous T2D association studies in Arabs.**

| **SNP** | **Gene** | **Effect Allele** | **Effect Size (CI)** | **P-value** |
| --- | --- | --- | --- | --- |
| **Study 1:** Al-Daghri et al^1^ used 1235 non-diabetic and 1166 diabetic adult Saudi Arabian individuals to study 38 SNPs previously implicated for T2D. **Replicated established risk variants and loci.** | | | | |
| rs1801214 | *WFS1* | T | 1.26 (1.09-1.46) | 1.63E-03 |
| rs849134 | *JAZF1* | A | 1.20 (1.04-1.39) | 2.00E-02 |
| rs10965250 | *CDKN2A/B* | G | 1.22 (1.03-1.44) | 2.00E-02 |
| rs7903146 | *TCF7L2* | T | 1.55 (1.33-1.80) | 1.13E-08 |
| rs231362 | *KCNQ1* | G | 1.17 (1.02-1.35) | 3.00E-02 |
| rs163184 | *KCNQ1* | G | 1.16 (1.01-1.33) | 4.00E-02 |
| rs4812829 | *HNF4A* | A | 1.27 (1.07-1.51) | 6.80E-03 |
| rs5945326 | *DUSP9* | A | 1.34 (1.04-1.73) | 2.00E-02 |
| **Study 2:** O’Beirne et al^2^ used ≥3 generation of Qatari adults with age >30; cohort comprised 1124 diabetics and 590 non-diabetics to study 37 SNPs previously implicated for T2D along with additional 27 tag SNPs. **Replicated established risk variants and loci.** | | | | |
| rs4506565 | *TCF7L2* | T | 1.33 (1.12-1.58) | 3.70E-02 |
| rs7903146 | *TCF7L2* | T | 1.36 (1.14-1.62) | 2.90E-02 |
| **Study 3:** O’Beirne et al^3^ used ≥3 generation of Qatari adults with age >30; cohort comprised 574 diabetics and 290 non-diabetics to study exome-wide (295,515) SNPs associations; SKAT analysis was performed. **Exome-wide association analysis. Identified novel risk variants and loci.** | | | | |
| Chr9:114091623-114100099 (chr9:116859679) | *KIF12* |  |  | 2.37E-09 |
| Chr1:1335278-1349142 (chr1:1271676) | *DVL1* |  |  | 3.30E-07 |
| Chr18:5392381-5630666 (chr18:5397367, rs8082898, rs117900256) | *EPB41L3* |  |  | 9.91E-07 |
| Chr2:25377220-25673647 (chr2:25611134, rs562264712) | *DTNB* |  |  | 1.20E-06 |
| Chr6:170282200-170291075 (rs200861263) | *DLL1* |  |  | 3.34E-06 |
| Chr3:41199451-41240448 (rs77750814) | *CTNNB1* |  |  | 3.35E-06 |
| **Study 4:** Dajani et al^4^ used 67 diabetics and 214 non-diabetics of Jordan subpopulation (such as Chechan and Circassian); performed regional imputation and logistic regression to study **genome-wide SNP association analysis**. **Identified novel risk variants and loci.** | | | | |
| rs6134031 | *JAG1* |  | 9.66 | 1.12E-08 |
| rs4758690 | *MLXIP* |  | 3.00 | 4.20E-05 |
| **Study 5:** Ghassibe-Sabbagh et al^5^ used 1902 non-diabetics and 1384 diabetics of Lebanese individuals; performed imputation and logistic regression (adjusted for age,sex and BMI) analysis to study **genome-wide SNP association analysis**. **Novel and established** r**isk variants from established risk loci.** | | | | |
| rs7766070 | *CDKLAL1* |  | 1.39(1.25-1.55) | 4.77E-09 |
| rs9348441 | *CDKLAL1* |  | 1.39(1.24-1.55) | 6.38E-09 |
| rs35261542 | *CDKLAL1* |  | 1.39(1.24-1.55) | 1.45E-08 |
| rs7451008 | *CDKLAL1* |  | 1.39(1.24-1.55) | 1.31E-08 |
| rs1569699 | *CDKLAL1* |  | 1.35(1.22-1.49) | 1.49E-08 |
| rs9368222 | *CDKLAL1* |  | 1.38(1.23-1.53) | 1.65E-08 |
| rs9350271 | *CDKLAL1* |  | 1.34(1.21-1.49) | 2.59E-08 |
| rs10946396 | *CDKLAL1* |  | 1.34(1.21-1.48) | 4.08E-08 |
| rs6456369 | *CDKLAL1* |  | 1.33(1.2-1.48) | 5.20E-08 |
| rs6456368 | *CDKLAL1* |  | 1.35(1.21-1.5) | 6.91E-08 |
| rs7754840 | *CDKLAL1* |  | 1.35(1.21-1.5) | 6.71E-08 |
| rs4710940 | *CDKLAL1* |  | 1.33(1.2-1.48) | 6.28E-08 |
| rs35456723 | *CDKLAL1* |  | 1.35(1.21-1.5) | 7.44E-08 |
| rs9295474 | *CDKLAL1* |  | 1.36(1.22-1.51) | 4.51E-08 |
| rs7756992 | *CDKLAL1* |  | 1.35(1.22-1.51) | 4.33E-08 |
| rs6456367 | *CDKLAL1* |  | 1.35(1.21-1.5) | 7.07E-08 |
| chr6:20660689 | *CDKLAL1* |  | 1.33(1.2-1.48) | 7.54E-08 |
| rs7752780 | *CDKLAL1* |  | 1.35(1.21-1.5) | 7.31E-08 |
| rs34872471 | *TCF7L2* |  | 1.35(1.22-1.5) | 1.01E-08 |
| rs35198068 | *TCF7L2* |  | 1.35(1.22-1.49) | 1.42E-08 |
| rs7903146 | *TCF7L2* |  | 1.34(1.21-1.49) | 1.79E-08 |
| rs4506565 | *TCF7L2* |  | 1.34(1.21-1.48) | 1.96E-08 |
| rs7901695 | *TCF7L2* |  | 1.33(1.2-1.47) | 4.50E-08 |
| **Study 6:** Al Safar et al^6^ used 282 diabetic and 311 non-diabetics of UAE extended family individuals to perform **family-based genome-wide association analysis**. **Identified novel risk variants from novel risk loci.** | | | | |
| rs7675224 | *KCTD8* |  |  | 1.20E-3 |
| rs4407541 | *KCTD8* |  |  | 4.00E-4 |
| rs4695718 | *KCTD8* |  |  | 2.00E-4 |
| rs7692404 | *GABRG1* |  |  | 3.10E-3 |
| rs1353642 | *GABRG1* |  |  | 6.59E-5 |
| rs279856 | *GABRA2* |  |  | 4.00E-04 |
| rs7679715 | *COX7B2* |  |  | 1.83E-05 |
| rs2055942 | *GABRA4* |  |  | 3.00E-4 |
| rs1953722 | *PRKD1* |  |  | 4.00E-04 |
| **Study 7:** Hebbar et al^7^ used 536 diabetics and 1429 non-diabetics of Arab ethnics and performed quantitative trait analysis (HbA1c and FBG) to study genome-wide SNP association**. Identified** n**ovel risk variants from novel risk loci.** | | | | |
| rs12440118 | *ZNF106* (W > R) | G | 1.498 | 2.70E-05 |
| rs7144734 | *OTX2-AS1* | A | 1.361 | 4.31E-07 |

**References**

1. Al-Daghri , NM. *et al.* Assessing the contribution of 38 genetic loci to the risk of type 2 diabetes in the Saudi Arabian population. *Clin Endocrinol 80,* 532 (2014) doi: 10.1111/cen.12187.

2. O’Beirne, SL. *et al.* Type 2 diabetes risk allele loci in the qatari population. *PLoS ONE 11,* e0156834 (2016) doi: 10.1371/journal.pone.0156834.

3. O'Beirne, SL. *et al.* Exome sequencing-based identification of novel type 2 diabetes risk allele loci in the Qatari population. *PLoS ONE 13,* e0199837 (2018) doi: 10.1371/journal.pone.0199837.

4. Dajani, R. *et al.* Genome-wide association study identifies novel type II diabetes risk loci in Jordan subpopulations. *PeerJ 5*, e3618 (2017) doi:10.7717/peerj.3618.

5. Ghassibe-Sabbagh, M. *et al.* T2DM GWAS in the Lebanese population confirms the role of TCF7L2 and CDKAL1 in disease susceptibility. *Sci Rep* *4*, 7351 (2014) doi: 10.1038/srep07351.

6. Al Safar, HS. *et al.* A genome-wide search for type 2 diabetes susceptibility genes in an extended Arab family. *Ann Hum Genet 77*, 488-503 (2013) doi: 10.1111/ahg.12036.

7. Hebbar, P. *et al.* Genetic risk variants for metabolic traits in Arab populations. *Sci Rep* 7, 40988 (2017) doi:10.1038/srep40988.

**Table S6. Comparison of genotype distributions at the identified risk variants between the Arab population and continental populations.**

| SNP | Population | MAF | Reference homozygous genotype | Alternate heterozygous genotype | Alternate homozygous genotype | P-values for difference in genotype distributions between the Arab population and continental populations^$^. |
| --- | --- | --- | --- | --- | --- | --- |
| rs1002487 | Arab | 0.057 | 0.883 | 0.111 | 0.003 |  |
|  | AFR | 0.042 | 0.915 | 0.085 | 0 | **0.02** |
|  | AMR | 0.032 | 0.937 | 0.063 | 0 | **5.3e-05** |
|  | EAS | 0.13 | 0.758 | 0.224 | 0.018 | **5.08e-14** |
|  | EUR | 0.065 | 0.875 | 0.121 | 0.004 | 0.730 |
|  | SAS | 0.089 | 0.838 | 0.115 | 0.016 | **0.006** |
|  | ALL | 0.072 | 0.863 | 0.129 | 0.008 | 0.153 |
|  |  |  |  |  |  |  |
| rs707927 | Arab | 0.1062 | 0.797 | 0.189 | 0.011 |  |
|  | AFR | 0.148 | 0.725 | 0.256 | 0.02 | **3.52e-05** |
|  | AMR | 0.069 | 0.862 | 0.138 | 0 | **5.63e-06** |
|  | EAS | 0.122 | 0.771 | 0.216 | 0.014 | 0.265 |
|  | EUR | 0.03 | 0.942 | 0.056 | 0.002 | **<2.2e-16** |
|  | SAS | 0.054 | 0.902 | 0.088 | 0.01 | **1.06e-12** |
|  | ALL | 0.09 | 0.831 | 0.159 | 0.01 | **<2.2e-16** |
|  |  |  |  |  |  |  |
| rs487321 | Arab | 0.0821 | 0.006 | 0.152 | 0.843 |  |
|  | AFR | 0.054 |  | 0.107 | 0.893 | **0.00022** |
|  | AMR | 0.151 | 0.026 | 0.251 | 0.723 | **4.60e-11** |
|  | EAS | 0.028 | 0 | 0.056 | 0.944 | **8.69e-14** |
|  | EUR | 0.088 | 0.004 | 0.169 | 0.827 | 0.527 |
|  | SAS | 0.07 | 0.004 | 0.131 | 0.865 | 0.306 |
|  | ALL | 0.072 | 0.005 | 0.134 | 0.861 | 0.482 |
|  |  |  |  |  |  |  |
| rs12600570 | Arab | 0.1482 | 0.728 | 0.246 | 0.025 |  |
|  | AFR | 0.452 | 0.298 | 0.499 | 0.203 | **<2.2e-16** |
|  | AMR | 0.115 | 0.775 | 0.219 | 0.006 | **0.00053** |
|  | EAS | 0.174 | 0.683 | 0.288 | 0.03 | **8.17e-06** |
|  | EUR | 0.121 | 0.771 | 0.215 | 0.014 | 0.039 |
|  | SAS | 0.167 | 0.683 | 0.301 | 0.016 | **0.017** |
|  | ALL | 0.227 | 0.612 | 0.322 | 0.066 | **3.03e-09** |
|  |  |  |  |  |  |  |

**SUPPLEMENTARY FIGURES**

**Figure S1. Scatter plots representing the first three principal components of the discovery cohort and representative HGDP populations.** The dynamic view of the plot is available at <http://dgr.dasmaninstitute.org/tcn2_pca/index.html>. The reference populations include: West Asia: Negev Bedouin (from Israel, denoting Arabian ancestry), Druze (from Israel), Palestinian (from central region of Israel); Central and South Asia: Brahui tribe (Balochistan in Pakistan); Africa: Mozabite (from North Africa); Europe: French Basque. This figure is as reproduced from our previous publication of [Hebbar et al (2017) The TCN2 variant of rs9606756 [Ile23Val] acts as risk loci for obesity-related traits and mediates by interacting with Apo-A1. *Obesity (Silver Spring)* **25**: 1098-1108].


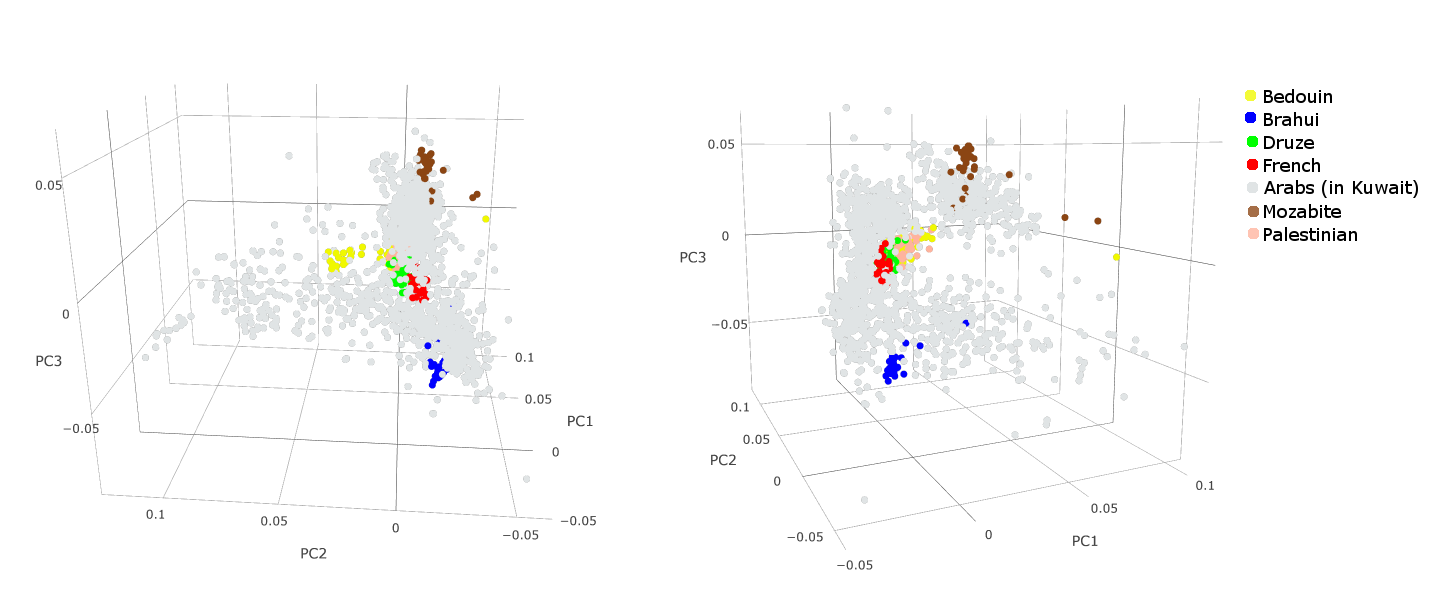


**Figure S2. Published PCA plot for native Kuwaitis of Arab ethnicity confirmed through detailed surname lineage analysis.** This figure is as reproduced from our previous publication of [Alsmadi et al. (2013) Genetic substructure of Kuwaiti population reveals migration history. *PLoS One* **8**: e74913].


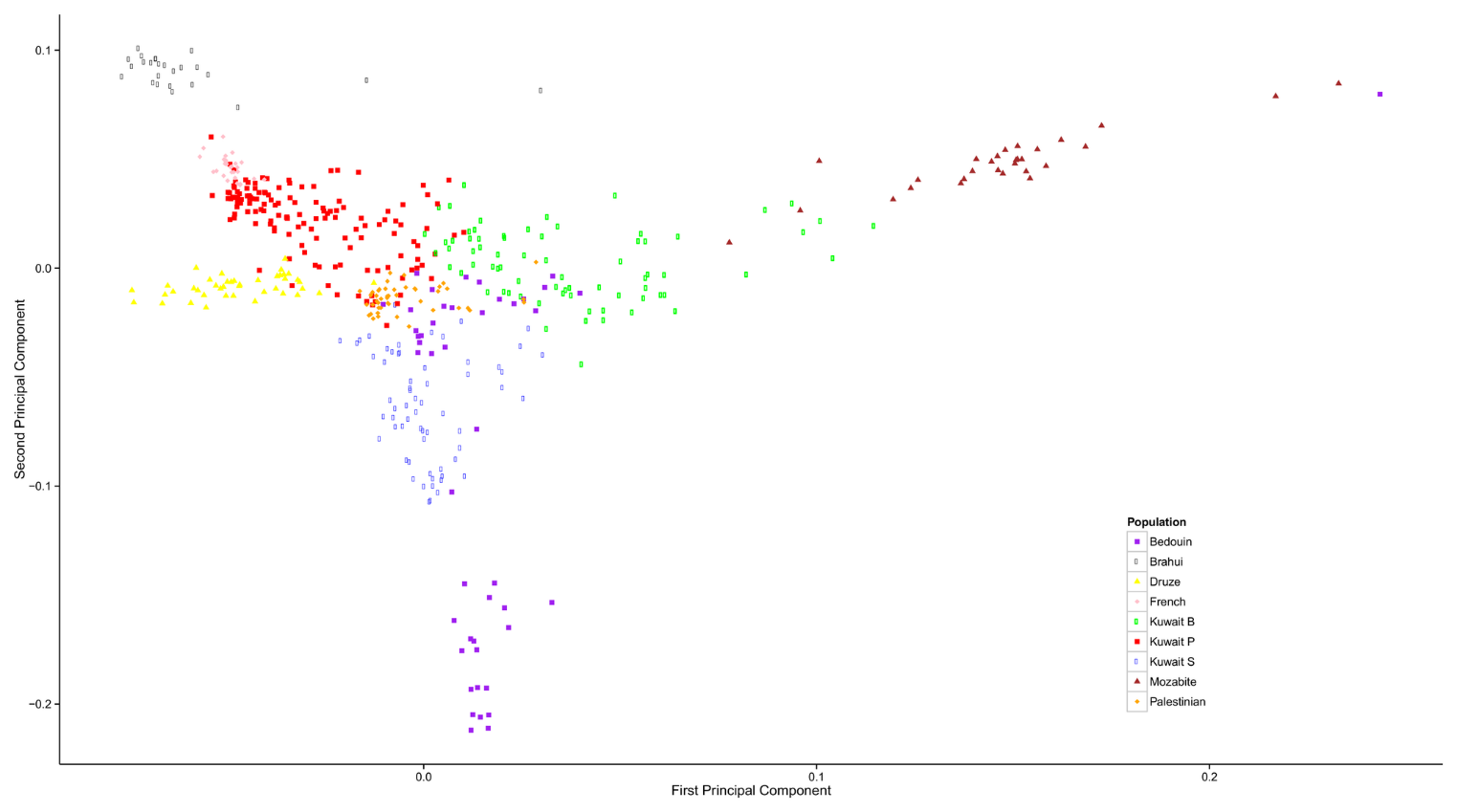


**Figure S3. Intensity maps** **for the 22 markers (selected for replication phase).** The plots depict the observed genotype clusters. The three highlighted clusters in each of the plots display the quality of the three different genotypes called - homozygous allele A (red), heterozygous AB (purple) and homozygous allele B (blue) – in the three genotyping arrays used to genotype the discovery cohort. The 22 markers are: rs1002487; rs4143782; rs12488539; rs6762914; rs487321; rs17065898; rs707927; rs1145784; rs2522219; rs1199028; rs2599723; rs3812689; rs918988; rs1151501; rs11179003; rs7329697; rs4646213; rs3784240; rs1256826; rs930514; rs12600570; rs9959376.

| **Batch 1** | **Batch 2** | **Batch 3** |
| --- | --- | --- |
| **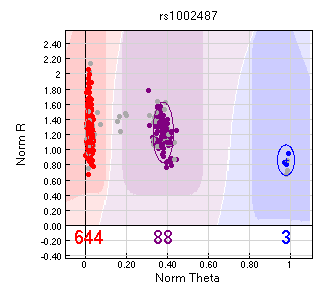** | **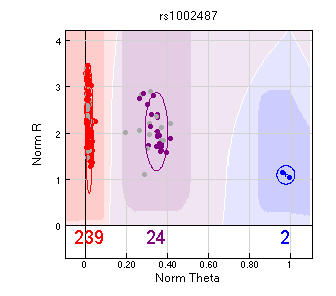** | **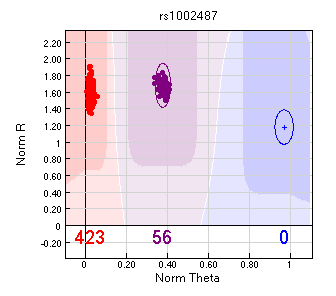** |
| **rs1002487** | **rs1002487** | **rs1002487** |
| 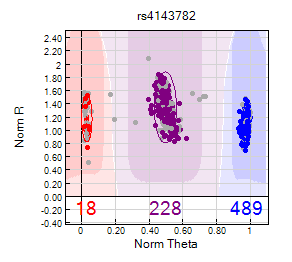 | 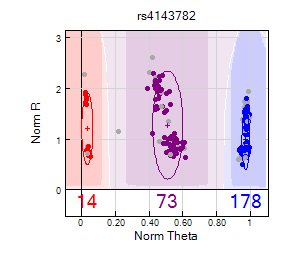 | 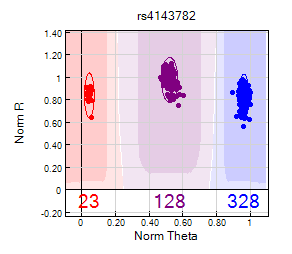 |
| **rs4143782** | **rs4143782** | **rs4143782** |
| 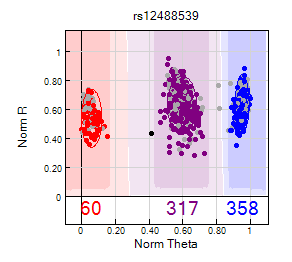 | 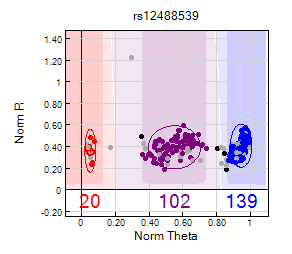 | 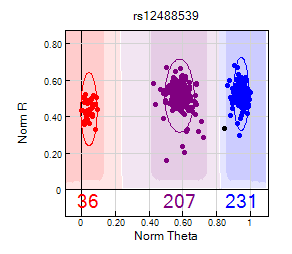 |
| **rs12488539** | **rs12488539** | **rs12488539** |
| 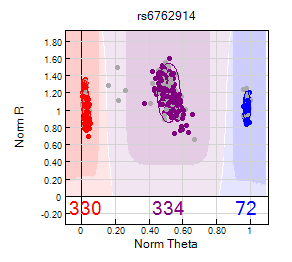 | 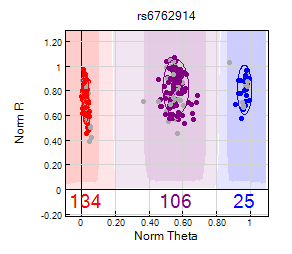 | 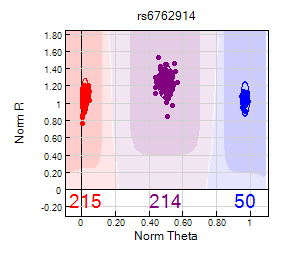 |
| **rs6762914** | **rs6762914** | **rs6762914** |
| 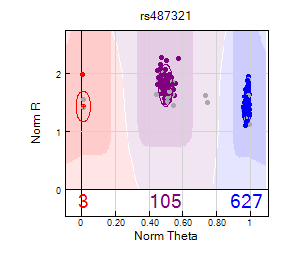 | 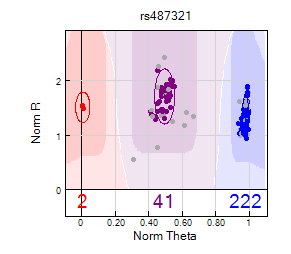 | 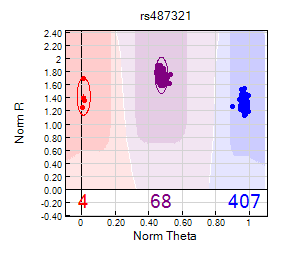 |
| **rs487321** | **rs487321** | **rs487321** |

| 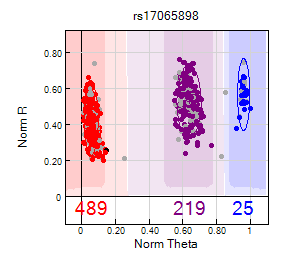 | 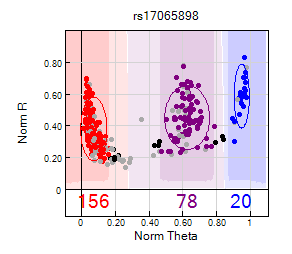 | 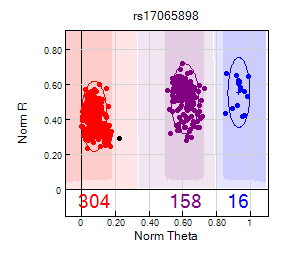 |
| --- | --- | --- |
| **rs17065898** | **rs17065898** | **rs17065898** |
| 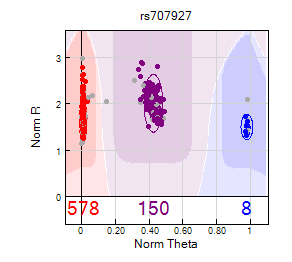 | 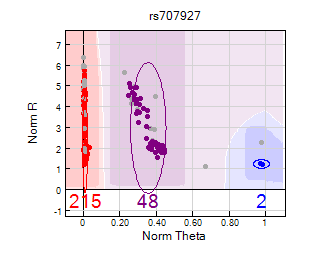 | 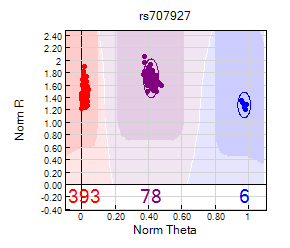 |
| **rs707927** | **rs707927** | **rs707927** |
| 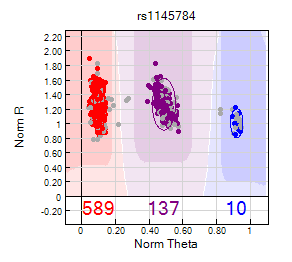 | 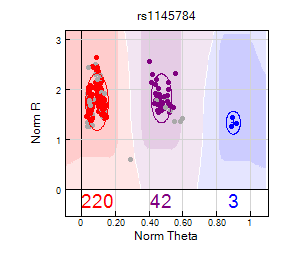 | 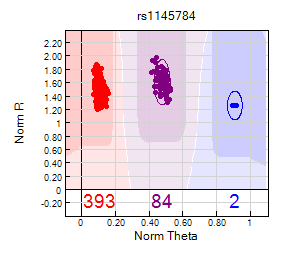 |
| **rs1145784** | **rs1145784** | **rs1145784** |

| 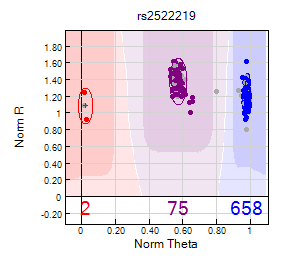 | 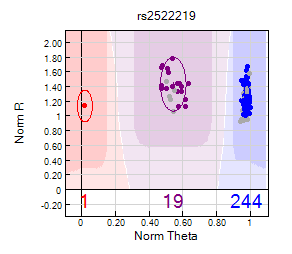 | 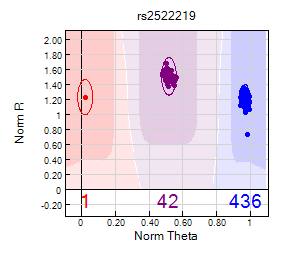 |
| --- | --- | --- |
| **rs2522219** | **rs2522219** | **rs2522219** |
| 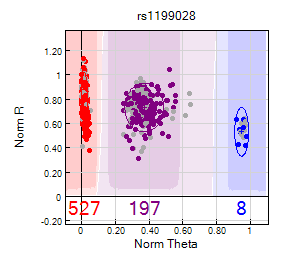 | 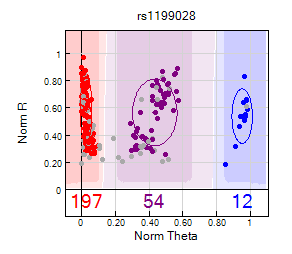 | 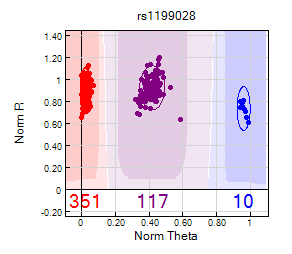 |
| **rs1199028** | **rs1199028** | **rs1199028** |
| 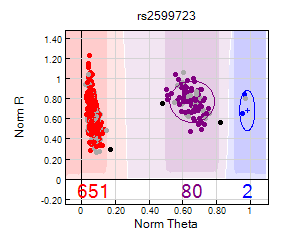 | 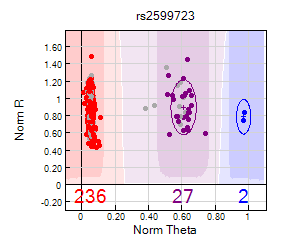 | 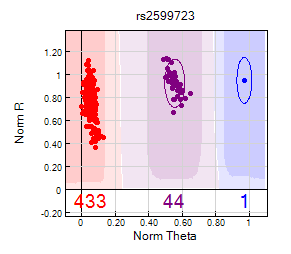 |
| **rs2599723** | **rs2599723** | **rs2599723** |
|  |  |  |
| 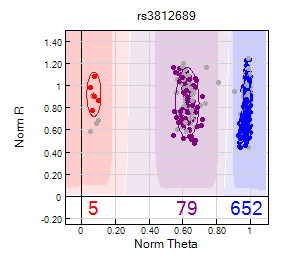 | 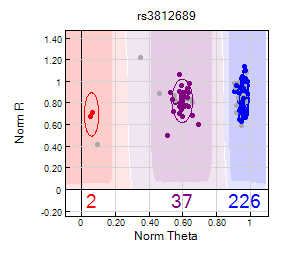 | 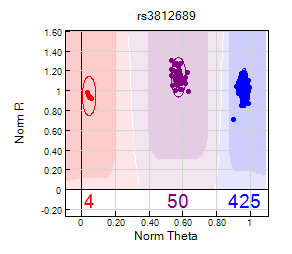 |
| **rs3812689** | **rs3812689** | **rs3812689** |
| 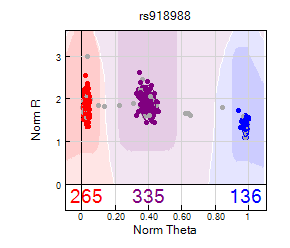 | 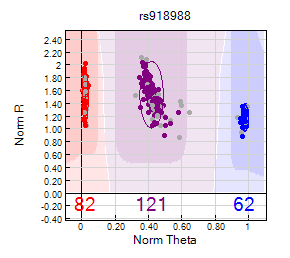 | 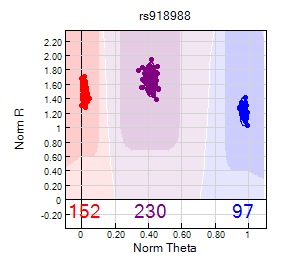 |
| **rs918988** | **rs918988** | **rs918988** |
| 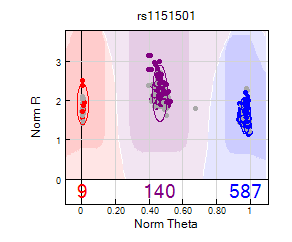 | 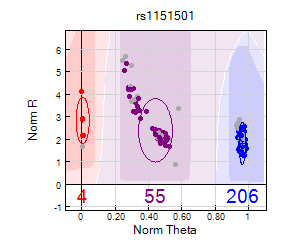 | 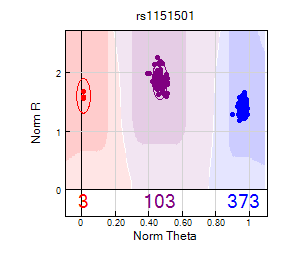 |
| **rs1151501** | **rs1151501** | **rs1151501** |
| 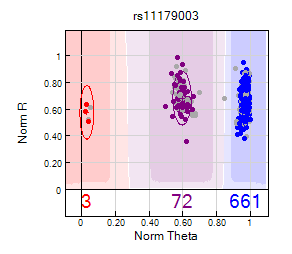 | 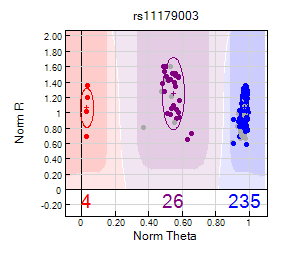 | 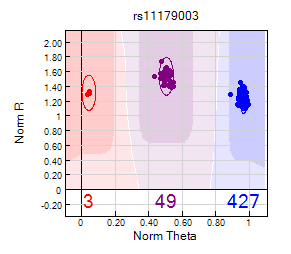 |
| **rs11179003** | **rs11179003** | **rs11179003** |
|  |  |  |
| 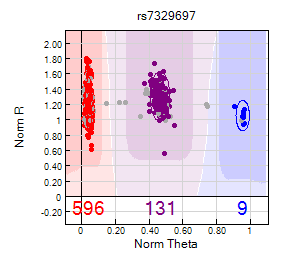 | 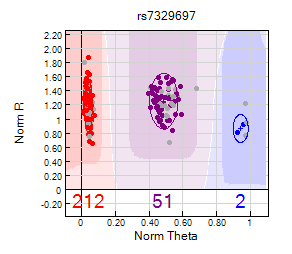 | 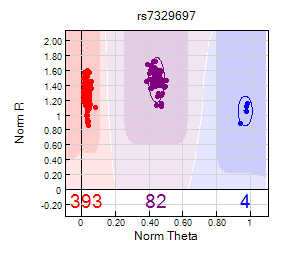 |
| **rs7329697** | **rs7329697** | **rs7329697** |
| 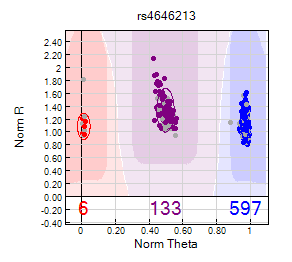 | 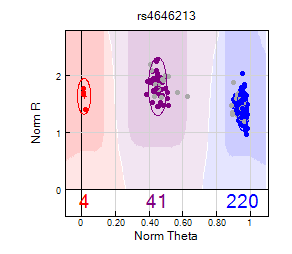 | 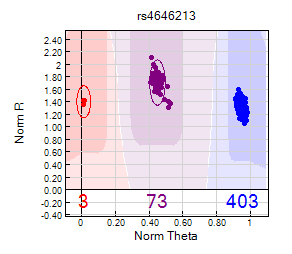 |
| **rs4646213** | **rs4646213** | **rs4646213** |
| 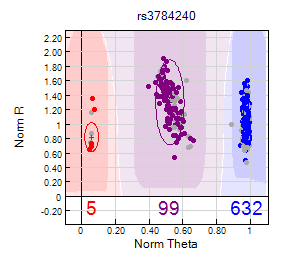 | 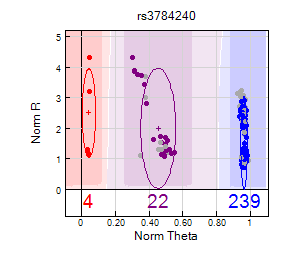 | 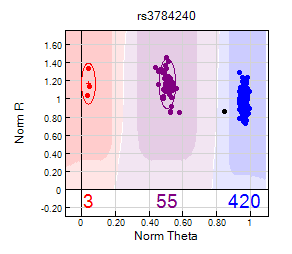 |
| **rs3784240** | **rs3784240** | **rs3784240** |
| 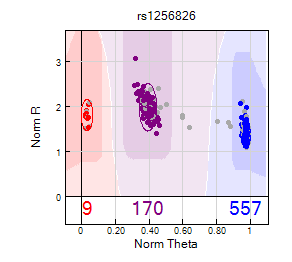 | 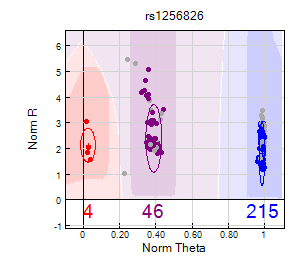 | 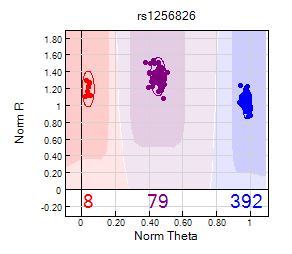 |
| **rs1256826** | **rs1256826** | **rs1256826** |
| 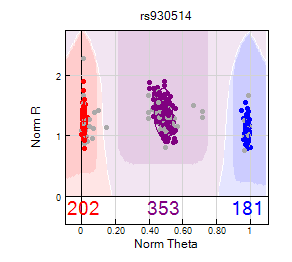 | 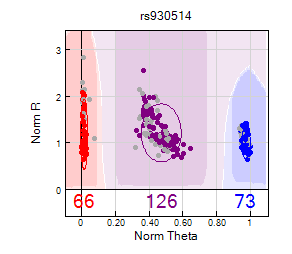 | 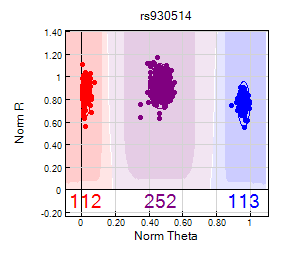 |
| **rs930514** | **rs930514** | **rs930514** |
| 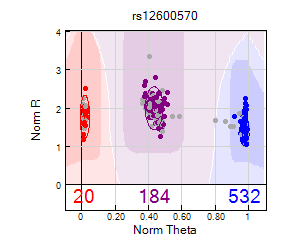 | 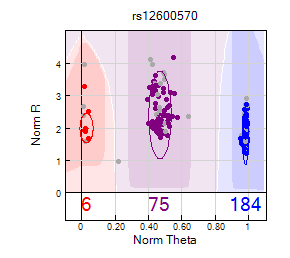 | 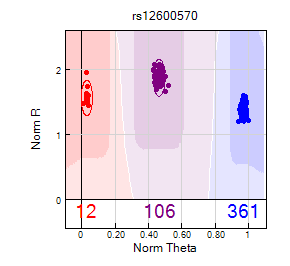 |
| **rs12600570** | **rs12600570** | **rs12600570** |
| 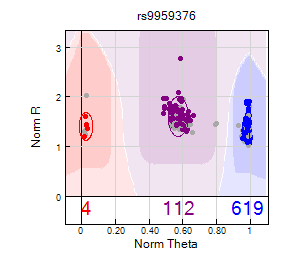 | 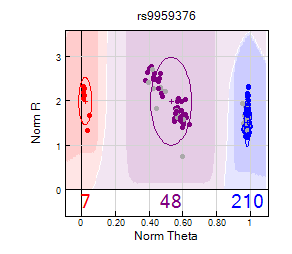 | 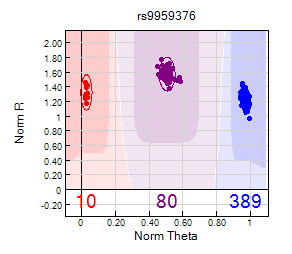 |
| **rs9959376** | **rs9959376** | **rs9959376** |

**Figure S4. Manhattan plots of the 632,375 SNPs employed to interrogate the genomes of the study population for associations with the FPG trait, under recessive or additive mode of inheritance.** Labelling is done by considering the threshold of P<1.84E-08 for genome wide significance.

1. **Recessive Model**


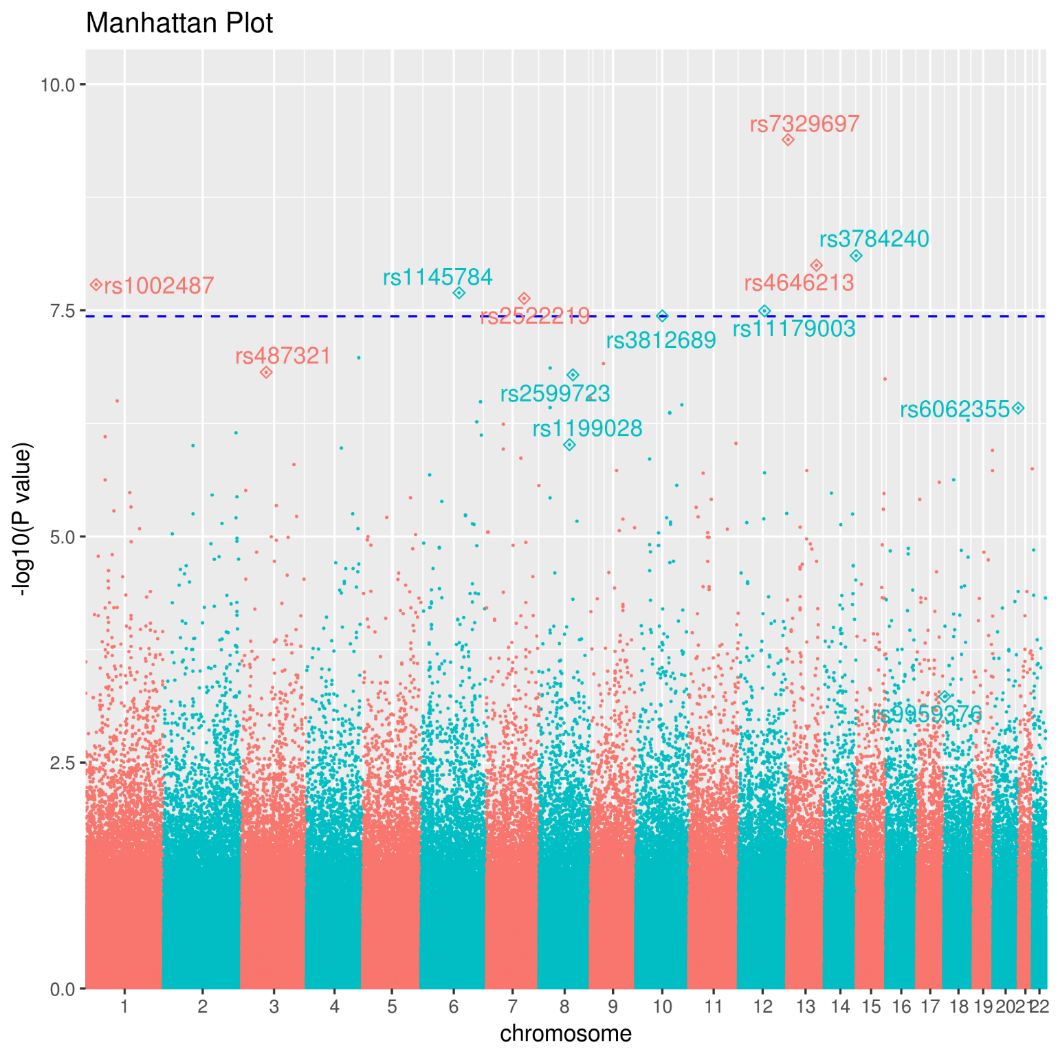


1. **Additive Model**


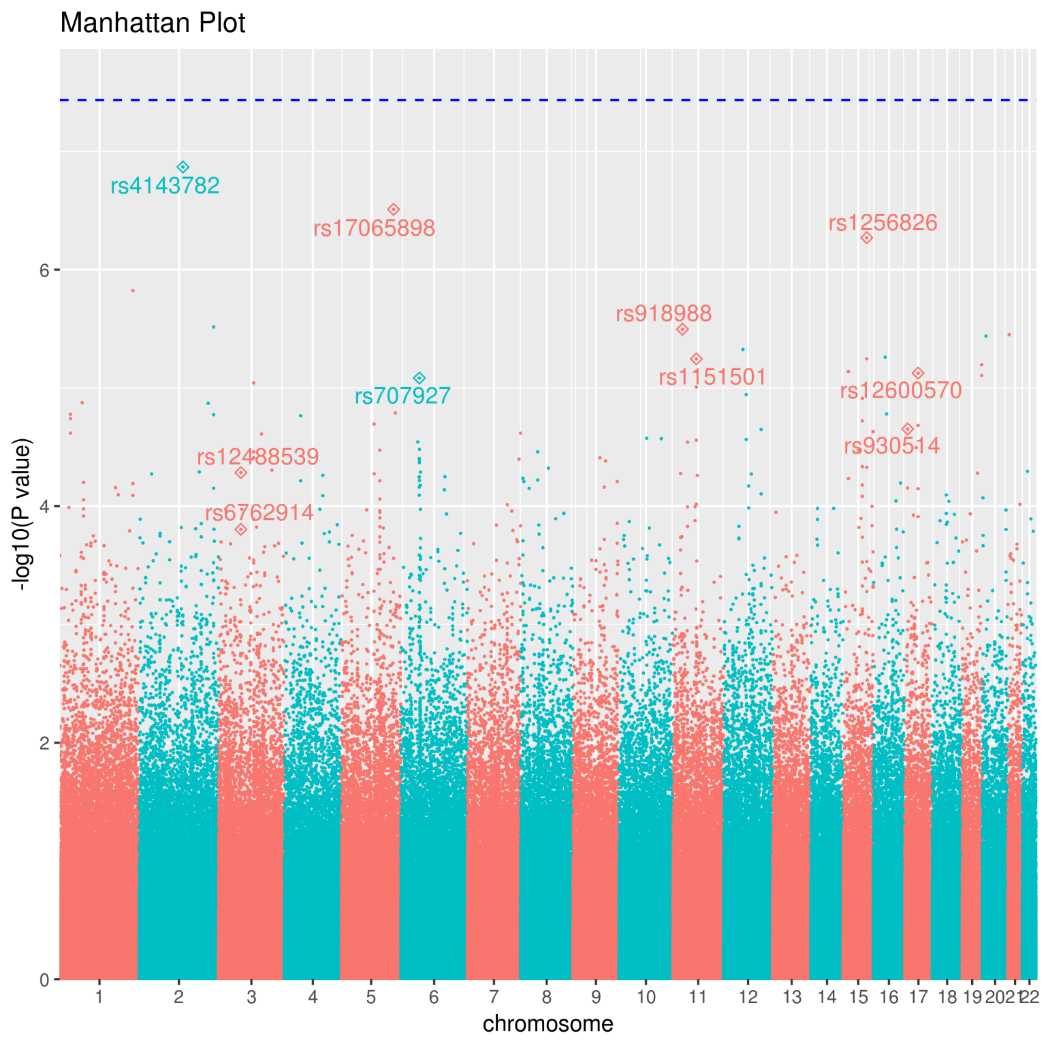


**Supplementary Figure S5. Comparison of risk allele frequencies at the identified risk variants across populations.**


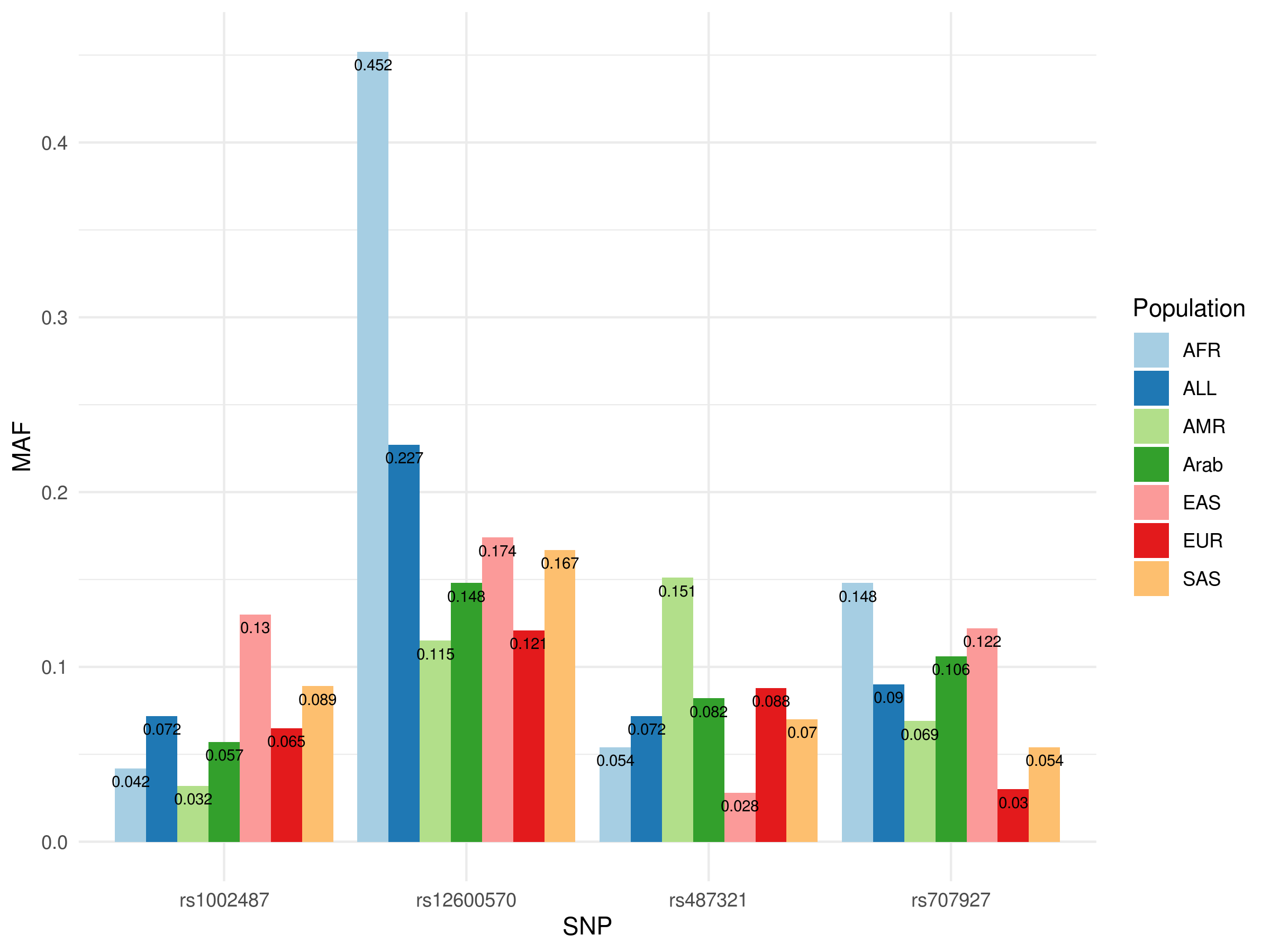

Supplement: Supplementary file 1 — Supplementary Material. [file 41598_2019_57072_MOESM1_ESM.docx]
